# Superior‐Selective and Complete Recycling of Trace Precious Metals From Wastewater by Magnetic Trilayer Carbon‐Aerogels

**DOI:** 10.1002/advs.202500858

**Published:** 2025-05-28

**Authors:** Jianzheng Yang, Yan Zhou, Shang Du, Bing Wu, Jianying Zhang, Shanjun Song, Tao Zhou, Jinming Zhang

**Affiliations:** ^1^ National Institute of Metrology Beijing 100013 China; ^2^ CAS Key Laboratory of Engineering Plastics Institute of Chemistry Chinese Academy of Sciences (CAS) Beijing 100190 China

**Keywords:** biomass, precious metals, resource recovery, selectivity, water treatment

## Abstract

It is a considerable challenge to selectively recycle precious metals from industrial wastewater and natural waters. Herein, carbon aerogels embedded with magnetic Fe@FeS@C nanoparticles (CFeS aerogels) were constructed from natural carrageenan. The unique redox potential of FeS, coupled with the barrier effect and the electron conduction property of the carbon layer, made the Fe@FeS@C nanoparticles exhibited the ultra‐high ion selectivity. The resultant CFeS aerogels can selectively adsorb and reduce trace Au(III), Ag(I), and Pd(II) ions at ppb level in an aqueous solution with 29 coexisting cations. Even if the concentration of competing metal ions is 100‐fold higher than those of the precious metal ions, the selectivity for Au(III), Ag(I), and Pd(II) ions remained above 99.2%. Moreover, the 3D carbon network immobilizing the Fe@FeS@C nanoparticles prevented the aggregation and detachment of the recycled precious metals, thereby enhancing the adsorption rate and capacity. CFeS aerogels rapidly achieved adsorption equilibrium for Au(III), Ag(I), and Pd(II) ions in 5–10 min, and have the saturated adsorption capacities of 321.2 mg·g^−1^, 150.6 mg·g^−1^, and 70.1 mg·g^−1^, respectively. Such aerogels with ultra‐high selectivity, high efficiency, and easy separation provided a practical strategy for the enrichment and recovery of the precious metals.

## Graphical abstract

Carbon aerogels embedded with magnetic Fe@FeS@C nanoparticles are fabricated to achieve a rapid and ultra‐highly selective recovery of the precious metals

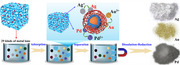

## 1 Introduction

Precious metals, such as gold, silver, and palladium, are scarce resources and have unique physicochemical properties, including high stability, catalytic activity, antimicrobial properties, and electrical conductivity, thus, they are extensively utilized in jewelry, chemical synthesis, biomedicine, and electronic equipment.^[^ ^1^, ^2^, ^3^ ^]^ With the development of modern industries, especially electronics, medical, and chemical industries, the consumption of precious metals has dramatically increased. The annual global consumption of gold, silver, and palladium amounts to 250 t, 12800 t, and 40 t, respectively, just in the electronics industry.^[^ ^4^ ^]^ However, during the mining, using, and recycling processes, substantial wastewater containing these precious metals with low concentrations was inevitably generated.^[^ ^5^ ^]^ In addition, in the river water and seawater, there are a large amount of precious metals with low concentrations.^[^ ^6^ ^]^ For instance, it is estimated that the oceans contain ≈20 million tons of gold, which vastly surpasses the total amount of gold extracted from terrestrial mines.^[^ ^7^ ^]^ Therefore, the extraction and recovery of precious metals from industrial effluents and natural waters is of significant importance. However, due to the super‐low concentration of precious metals and the presence of various interfering metals with high concentrations, selective adsorption and separation of precious metals present considerable challenges.

To achieve the recovery of precious metals from water bodies, researchers have tried plenty of materials, such as porous organic polymers,^[^ ^8^ ^]^ metal‐organic frameworks,^[^ ^9^ ^]^ graphene,^[^ ^10^ ^]^ and bio‐derived materials.^[^ ^11^ ^]^ Li et al.^[^ ^12^ ^]^ utilized poly(ionic liquid)‐derived porous organic polycarbene to selectively adsorb Au(III) under the coexistence of Pt(II), Cu(II), Ni(II) and Zn(II). The adsorption amount was as high as 2.09 g·g^−1^. Chaudhuri et al.^[^ ^13^ ^]^ reported the effective adsorption of Pt(IV) and Pd(II) from highly acidic solution by using amino‐functionalized graphene oxide‐based dendritic adsorbent. Yang et al.^[^ ^14^ ^]^ recovered silver from electroplating wastewater by leveraging the selective reduction effect of mixed‐valence molybdenum oxide (MoO_x_) on Ag(I). However, despite the high specific surface area and tunable active sites of the aforementioned materials that enable superior adsorption capacity and selectivity, they still face challenges in practical applications, including complex synthesis, high costs, aggregation tendencies in acidic/aqueous environments, and poor stability. More importantly, the concentration of precious metal ions in actual water bodies is exceedingly low, typically at ppm level in industrial wastewater and at ppb level in natural waters, such as seawater.^[^ ^15^ ^]^ So, the absorbents which rely on the volume‐matching mechanism and weak‐interaction mechanism struggle to rapidly adsorb trace precious metal ions.^[^ ^16^ ^]^ Furthermore, the composition of real water bodies is complex and diverse, containing not only alkali metal ions, such as Na(I), K(I), Ca(II) and Mg(II), but also competing ions, like Co(II), Ni(II), Cu(II), Zn(II), Cd(II) and Pb(II), as well as rare‐earth metal ions. The concentrations of these common ions far exceed those of the target precious metal ions.^[^ ^7^ ^]^ The presence of interfering ions complicates the selective adsorption process of the absorbents that operate on the soft‐hard acid‐base principle, coordination effect, and volume effect, making it difficult to achieve a highly‐selective adsorption. Therefore, it remains a formidable challenge that the development of new materials capable of highly‐selective recovery of trace precious metals from complex environments.

**Figure 1:**
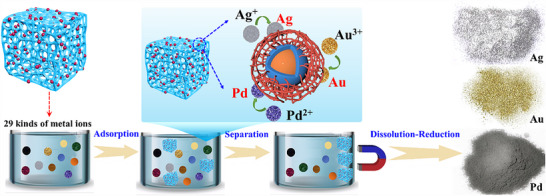
Schematic of selective adsorption of precious metal ions in an aqueous solution with 29 coexisting cations by using CFeS aerogels.

In this work, we demonstrated a new principle to realize rapid and ultra‐highly selective adsorption of precious metal ions by an interfacial selective‐reduction process. Natural carrageenan bearing sulfonate groups were used to fabricate carbon aerogels embedded with magnetic trilayer Fe@FeS@C nanoparticles (CFeS aerogels) via a simple and economical process. CFeS aerogels exhibited ultra‐high ion selectivity (**Figure** **1**), and could rapidly and selectively capture trace Au(III), Ag(I), and Pd(II) at ppb‐level concentrations in an aqueous solution with 29 coexisting cations.

## 2 Results and Discussion

### 2.1 Preparation of CFeS Aerogels

**Figure 2:**
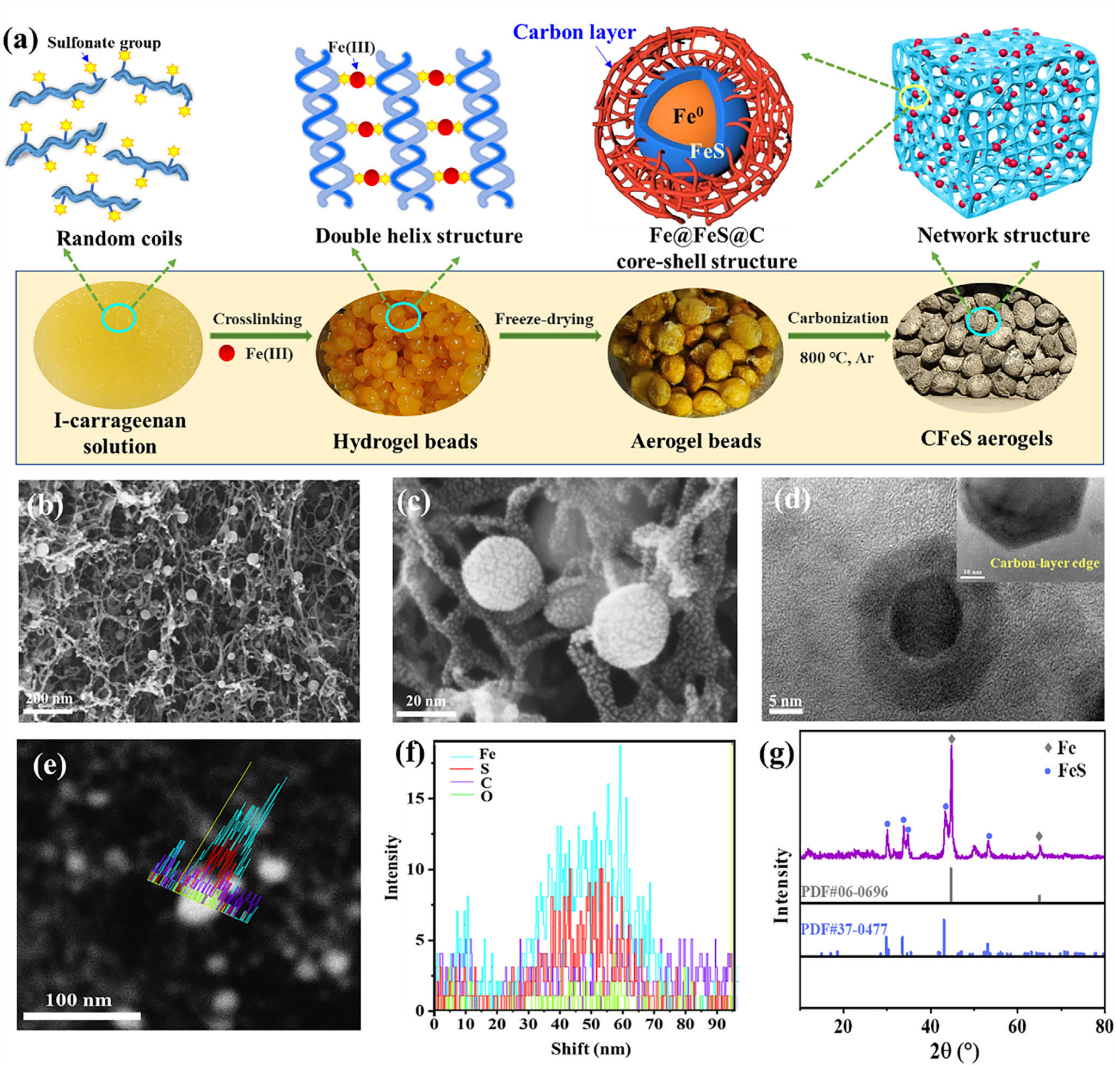
Fabrication of CFeS aerogels. a) Schematic diagram of CFeS aerogel fabrication. b) and c) SEM images of CFeS aerogel at different magnifications. d) TEM images of CFeS aerogel (The inset is a locally‐enlarged image of the edge). e) and f) TEM‐EDS line profiles of single Fe@FeS@C nanoparticle. g) XRD pattern of CFeS aerogel.

CFeS aerogels were fabricated via an ion‐induced gelation and subsequent carbonization process (**Figure** **2**a). Natural carrageenan bearing sulfonate groups was used as the raw materials. After dropping carrageenan aqueous solution into FeCl_3_ solution, carrageenan‐Fe(III) hydrogel spheres were obtained because of the strong coordination interactions between sulfonate groups and Fe(III) ions (Figure S1, Supporting Information). After a freeze‐drying step, carrageenan‐Fe(III) aerogel spheres were prepared (Figures S2 and S3, Supporting Information). Subsequently, via a high‐temperature pyrolysis under Ar atmosphere, the polymer skeleton was transformed into a porous carbon network, the Fe(III)‐sulfonate complex underwent a conversion to FeS, meanwhile a portion of the FeS was reduced to Fe^0^ by the carbon. Carbon aerogels embedded with magnetic Fe@FeS@C nanoparticles (CFeS aerogels) were fabricated (Figure 2b–g).

CFeS aerogel had a 3D interconnected porous network structure (Figure 2b,c). A large number of nanospheres were embedded inside the network, and were immobilized by the nanofibers. They were uniformly distributed without agglomeration. The N_2_ adsorption isotherms of the CFeS aerogel exhibited type I curve, indicating the presence of both micropores and nanopores (Figure S4, Supporting Information). The specific surface area was 387.7 m^2^·g^−1^. According to the particle size statistics, the nanospheres in the CFeS aerogel had an average diameter of 26 nm (Figure S5, Supporting Information). TEM images revealed that these nanospheres had a three‐layer core‐shell architecture, with a core of ≈10 nm in diameter, encased by an inner shell of ≈5 nm in thickness and an outer shell of 1–2 nm in thickness (Figure 2d). The EDS line‐scan analysis of a single core‐shell‐structured nanoparticle reveals that the nanoparticle comprises four elements, including Fe, S, C, and O (Figure 2e,f; S6, Supporting Information). Fe and S dominate the elemental composition. The iron content is significantly enriched in regions proximal to the core, while carbon exhibits a uniform spatial distribution. Furthermore, the total iron content in the nanoparticle substantially exceeds that of sulfur (Figure S6, Supporting Information). XRD curve of CFeS aerogel showed that there were the two sharp peaks at 44.7° and 65.2° which were assigned to the (110) and (200) crystal planes of Fe, respectively (Figure 2g). The diffraction peaks at 29.9°, 33.7°, 35.5°, 43.1° and 53.1° were corresponded to the (110), (112), (201), (114) and (300) crystal planes of FeS, respectively. Therefore, the core of the nanospheres was Fe, the inner shell layer was FeS, and the outer shell layer was C.

To summarize, the prepared CFeS aerogel has a distinctive string‐bead network structure, including carbon nanofibers and Fe@FeS@C nanoparticles. Carbon nanofibers acted as the structural framework, and Fe@FeS@C nanoparticles were immobilized on carbon nanofibers, effectively inhibiting the shedding and aggregation of Fe@FeS@C nanospheres. This special structure facilitatied the exposure of adsorption sites, thereby enhancing their adsorption efficiency and capacity. Fe@FeS@C nanospheres had a three‐layer structure, in which the core was magnetic Fe^0^, the indermediate layer was FeS, and the outer layer was carbon. Such innovative architecture endowed the Fe@FeS@C nanospheres with a robust resistance to acid and oxidizer, ensuring stable magnetic performance.

### 2.2 Adsorption Performance

**Figure 3:**
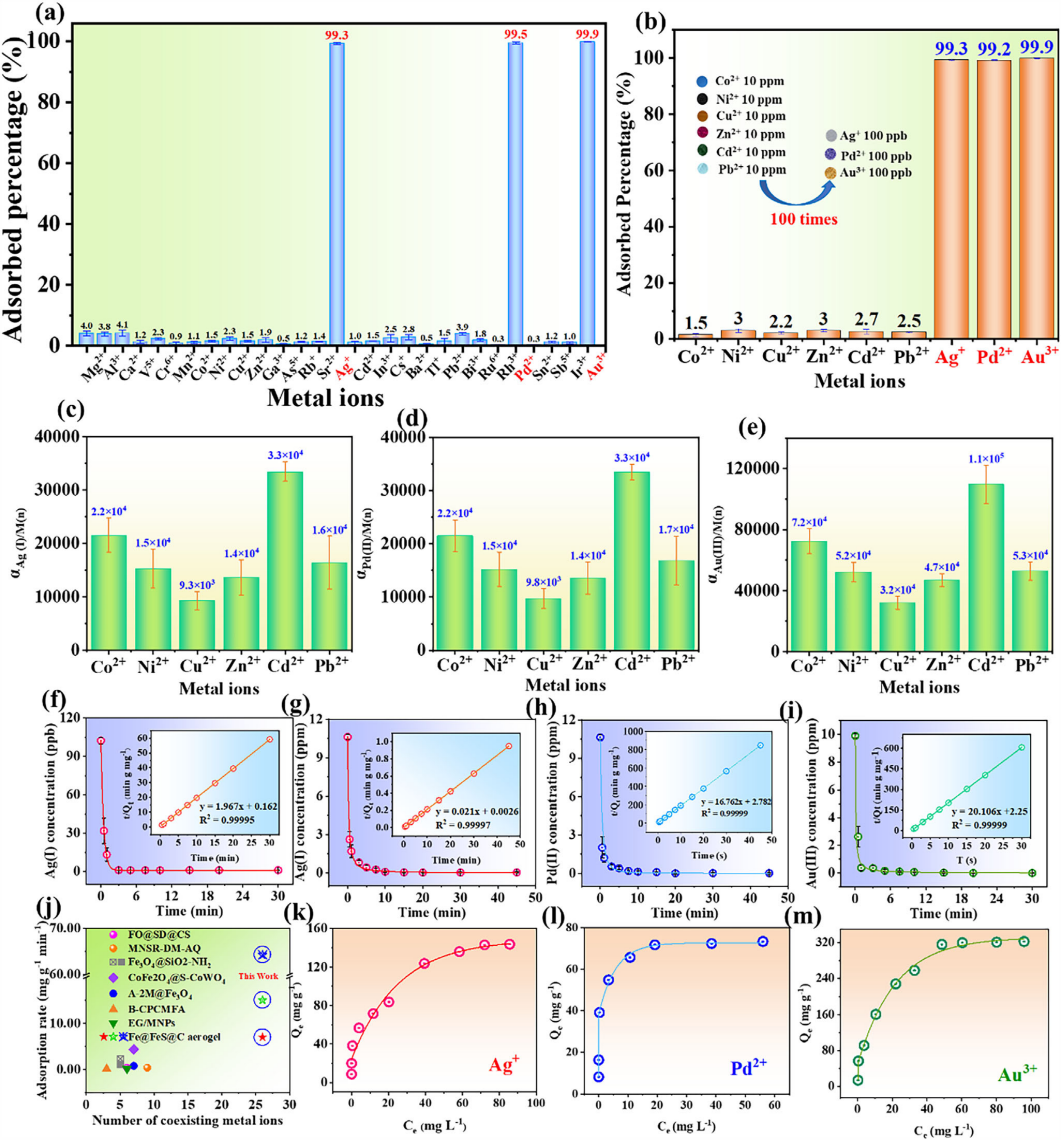
Adsorption performance of CFeS aerogels. a) Adsorption selectivity of CFeS aerogels among 29 metal ions (100 ppb per ion). b) Adsorption selectivity of CFeS aerogels among 9 metal ions. c) α_Ag(I)/M(n)_ values of different metal ions. d) α_Pd(II)/M(n)_ values of different metal ions. e) α_Au(III)/M(n)_ values of different metal ions. f) Adsorption kinetics of CFeS aerogels at an initial Ag(I) concentration of 100 ppb and the corresponding Pseudo‐second‐order kinetic plot. g) Adsorption kinetics of CFeS aerogels at an initial Ag(I) concentration of 10 ppm and the corresponding Pseudo‐second‐order kinetic plot. h) Adsorption kinetics of CFeS aerogels at an initial Pd(II) concentration of 10 ppm and the corresponding Pseudo‐second‐order kinetic plot. i) Adsorption kinetics of CFeS aerogels at an initial Au(III) concentration of 10 ppm and the corresponding Pseudo‐second‐order kinetic plot. j) Comparison of adsorption performance of representative adsorbents.^[^ ^27^, ^28^, ^29^, ^30^, ^31^, ^32^, ^33^ ^]^ k) Adsorption isotherm of Ag(I). l) Adsorption isotherm of Pd(II). (m) Adsorption isotherm of Au(III).

Selective adsorption of precious metal ions in the presence of interfering ions is the basis for the practical use of excellent adsorbent materials. A mixed solution containing 29 metal ions of Mg(II), Al(III), Ca(II), Cr(VI), Mn(II), Co(II), Ni(II), Cu(II), Zn(II), As(V), Ag(I), Cd(II), Pb(II), Pd(II) and Au(III), was configured to evaluate the adsorption selectivity of CFeS aerogels. Among them, Cu(II) (0.73 Å), Ni(II) (0.69 Å) and Zn(II) (0.74 Å), which have similar ionic radii to Pd(II) (0.66 Å) and Au(III) (0.66 Å), were considered from the site‐resistance effect.^[^ ^17^ ^]^ Based on the hard‐soft acid‐base theory, the hardness of Pb(II), Cd(II), Cu(II) Au(III), Pd(II), and Ag(I) are similar, all of which are soft acids or hard‐medial‐soft acids.^[^ ^18^, ^19^, ^20^ ^]^ From the viewpoint of coordination chemistry, Rh(III) and Ir(III), have similar coordination properties with Au(III).^[^ ^21^ ^]^ CFeS aerogels exhibited superior selectivity for the adsorption of precious metal ions, with the adsorption percentage of more than 99% for Ag(I), Pd(II), and Au(III) and less than 5% for other metal ions (**Figure** **3**a). Furthermore, the common metal ions in industrial water, including Co(II), Ni(II), Cu(II), Zn(II), Cd(II) and Pb(II), were chosen as interfering ions. The concentrations of the interfering ions (10 ppm) were 100 times higher than those of the Ag(I), Pd(II), and Au(III) (100 ppb). CFeS aerogel still maintained an ultra‐high adsorption selectivity, achieving an adsorption percentage exceeding 99% for Ag(I), Pd(II), and Au(III), while the absorption percentage for the interfering ions was less than 3% (Figure 3b). The selectivity coefficients (α_Ag(I)/M(n)_, α_Pd(II)/M(n)_ and α_Au(III)/M(n)_) of the CFeS aerogel consistently exceeded 10^4^ (Figure 3c–e), which was notably superior to those of previous materials, such as Bio‐MOF‐1 (α_Ag(I)/M(n)_ = 7 × 10^3^),^[^ ^22^ ^]^ COF‐SH (α_Ag(I)/Cu(II)_ = 2.7 × 10^2^, α_Ag(II)/Ni(II)_ = 1.2 × 10^3^, α_Ag(II)/Zn(II)_ = 4.0 × 10^3^),^[^ ^23^ ^]^ CuS nanoparticles (α_Pd(II)/Pb(II)_ = 8 × 10^3^, α_Pd(II)/Cd(II)_ = 1.2 × 10^4^),^[^ ^24^ ^]^ MXene hybrid aerogels (MXGA) (α_Au(III)/Pb(II)_ = 9.5 × 10^3^, α_Ag(I)/Co(II)_ = 5.4 × 10^2^, α_Ag(I)/Ni(II)_ = 3.0 × 10^3^),^[^ ^25^ ^]^ TpDa‐COF (α_Au(III)/M(n)_ = 1.2 × 10^2^).^[^ ^26^ ^]^ It is worth noting that in complex environments with high salt and humic acid, CFeS still exhibits highly selective adsorption of Ag(I), Pd(II), and Au(III) even in the presence of high concentrations of interfering ions (Figure S7, Supporting Information), demonstrating excellent selectivity and promising application prospects.

The unique string‐bead nanostructure and substantial specific surface area of CFeS aerogel conferred it with a remarkably‐rapid adsorption rate (Figure 3f–j and Table S1, Supporting Information). For the solution with initial Ag(I) concentration of 10 ppm and 100 ppb, the CFeS aerogel achieved over 99% adsorption within just 10 min and 3 min, respectively (Figure 3f,g), which was a ultra‐fast adsorption process and a high adsorption percentage. The affinity constant of CFeS aerogel for Ag(I), K_d_ = 5.3 × 10^5^ mL·g^−1^, met the criteria for an excellent adsorbent.^[^ ^34^ ^]^ Similarly, the CFeS aerogel rapidly captured more than 99% Pd(II) and Au(III). The Pd(II) adsorption rate was comparable to that of Ag(I), taking ≈10 min to reach equilibrium. Remarkably, the adsorption of Au(III) reached equilibrium more quickly, achieving a dramatic reduction from an initial concentration of 10 ppm to just 1 ppb in 5 min (Figure 3h–i). The corresponding K_d_ values of CFeS aerogel for Pd(II) and Au(III) were as high as 3.8 × 10^6^ mL·g^−1^ and 9.0 × 10^6^ mL·g^−1^, respectively. Consequently, CFeS aerogel possessed an outstanding capacity to effectively capture trace precious metal ions at ppb level. The adsorption kinetics data were fitted, revealing that the adsorption process for Ag(I), Pd(II), and Au(III) followed pseudo‐second‐order kinetics (Figure 3f–i).^[^ ^35^ ^]^ Thus, the adsorption process of CFeS aerogel for Ag(I), Pd(II), and Au(III) was chemisorption. The strong interaction between the adsorbent and adsorbate in chemisorption contributed to the rapid adsorption rate and ultra‐high adsorption percentage of the CFeS aerogel for Ag(I), Pd(II), and Au(III).

Isothermal adsorption experiments were conducted to assess the adsorption amount of CFeS aerogel for Ag(I), Pd(II) and Au(III). As the equilibrium concentration increased, the adsorption amount of the aerogel for these ions escalated swiftly, reaching maximum values of 150.6 mg·g^−1^ for Ag(I), 70.1 mg·g^−1^ for Pd(II), and 321.2 mg·g^−1^ for Au(III) (Figure 3k–m). Subsequently, the isothermal adsorption data were fitted using both Langmuir and Freundlich isotherm models (Figure S8 and Table S2, Supporting Information). The results indicated that the adsorption of Ag(I), Pd(II), and Au(III) by the CFeS aerogel aligned with the Langmuir isotherm, suggesting a monolayer adsorption process. This result was consistent with the conclusion of the kinetic fitting analysis that the adsorption process of CFeS aerogel for metal ions was chemisorption. Furthermore, a correlation with the Freundlich isotherm was observed also, perhaps because of the heterogeneous energy distribution across the adsorption sites. In the early stage of adsorption, monolayer adsorption mainly occurred, which was consistent with the Langmuir model. In the later stages of the adsorption, as the inhomogeneity of the surface adsorption increased, the energy difference of adsorption sites became pronounced, so the adsorption process followed the Freundlich model.^[^ ^36^, ^37^ ^]^

Additionally, the Fe^0^ core endowed the CFeS aerogel with strong magnetic property (Figure S9, Supporting Information). Thus, the CFeS aerogel could be rapidly separated by a magnetic separation process, facilitating its application in real world. In summary, the CFeS aerogel had exceptional adsorption selectivity, rapid uptake kinetics, substantial adsorption capacity, and easy separation, making it an ideal material to realize the adsorption and separation of precious metals in real water bodies.

To comprehensively evaluate the adsorption performance of Fe@FeS@C aerogel, we analyzed representative magnetic adsorbent materials reported over the past five years and compared critical metrics including adsorption rate, adsorption capacity, and adsorption selectivity (Table S1, Supporting Information). The results demonstrate that our material exhibits overwhelming advantages in adsorption rate and selectivity, while maintaining adsorption capacities comparable to observed materials. Collectively, these findings confirm that Fe@FeS@C aerogel is an exceptionally promising selective adsorption and separation material.

### 2.3 Selective Adsorption Mechanism

**Figure 4:**
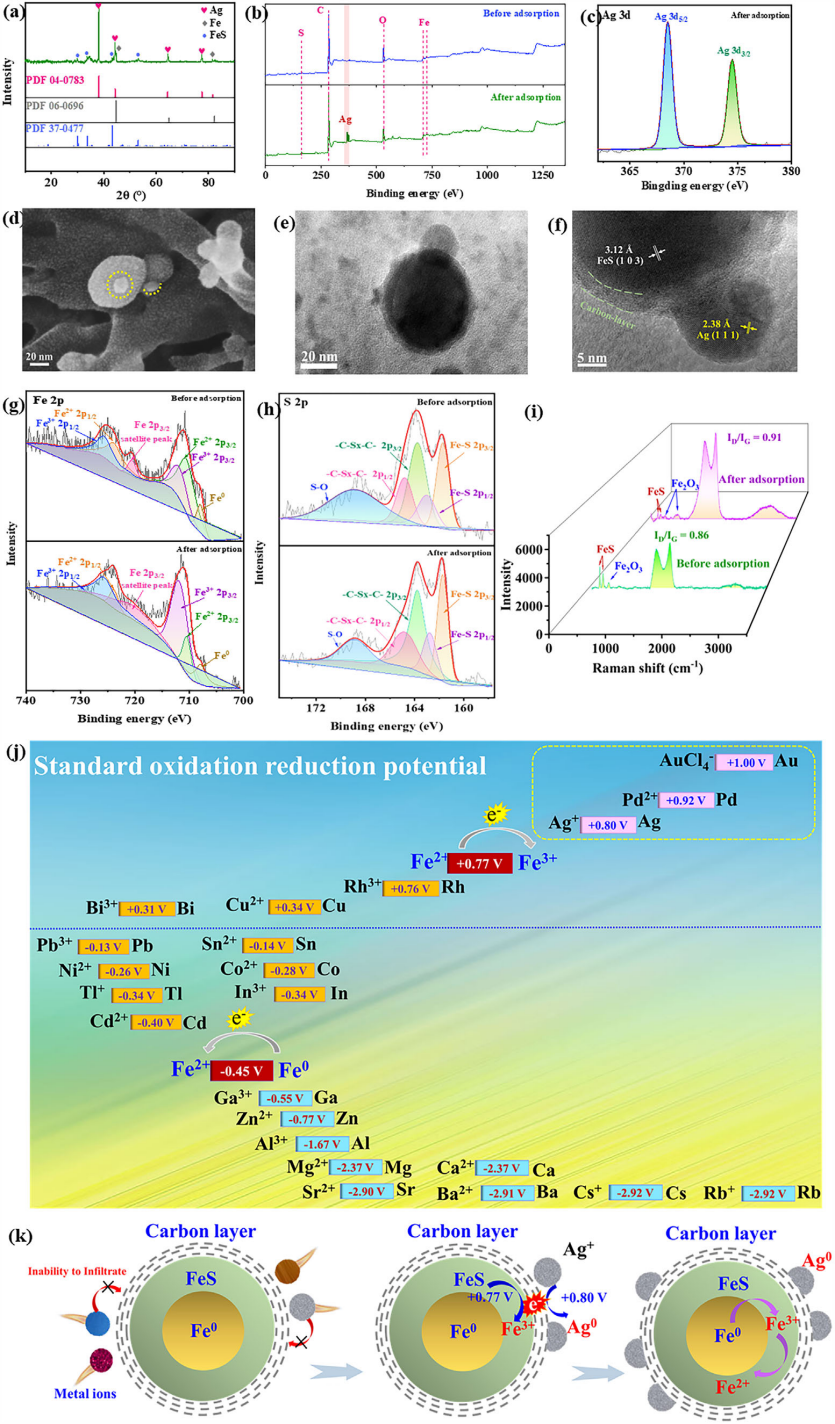
Selective adsorption mechanism. a) XRD pattern of CFeS aerogel after adsorption of Ag(I). b) XPS survey spectrum of CFeS aerogel before and after Ag(I) adsorption. c) High‐resolution Ag 3d XPS spectrum of CFeS aerogel after Ag(I) adsorption. d) SEM image, e) TEM image, and f) HR‐TEM image of CFeS aerogel after Ag(I) adsorption. High‐resolution g) Fe 2p and h) S 2p XPS spectra of CFeS aerogels before and after Ag(I) adsorption. i) Raman spectra of CFeS aerogel before and after Ag(I) adsorption. j) Standard redox potential ordering diagram. k) Schematic diagram of selective adsorption reduction of Ag(I) by CFeS aerogel.

The adsorption mechanism of CFeS aerogel was thoroughly investigated by taking the adsorption process of Ag(I) as an example. For the CFeS aerogel after adsorbing Ag(I), four new diffraction peaks located at 38.1°, 44.3°, 64.4° and 77.5° appeared in the XRD pattern, corresponding to the (111), (200), (220) and (311) crystal planes of Ag^0^, which indicated that Ag(I) had been reduced to Ag^0^ (**Figure** **4**a). In the XPS spectrum, the characteristic peaks of Ag appeared (Figure 4b). Two new peaks at 374.5 and 368.5 eV were attributed to the Ag 3d_5/2_ and Ag 3d_3/2_, respectively^[^ ^38^ ^]^ (Figure 4c). These results confirmed that the Ag(I) was transformed into Ag^0^ after using CFeS aerogel.

Subsequently, the elemental distribution of CFeS aerogel was observed after Ag(I) adsorption (Figure S10, Supporting Information). The Ag element was in close proximity to Fe and S elements, indicating that Ag(I) adsorption took place on the Fe@FeS@C nanoparticles, while the carbon nanofiber was not the adsorption site. This view was further supported by the FTIR spectra (Figure S11, Supporting Information). The IR absorption peaks of the carbon nanofiber network remained unchanged before and after the Ag(I) adsorption, such as the C─H stretching vibration peak (2910 cm^−1^), the C═O asymmetric stretching vibration peak (1640 cm^−1^), the C─O─C stretching vibration peak (1043 cm^−1^), and the S─O bending vibration peak (850 cm^−1^). SEM and TEM images provided direct evidence that Ag(I) adsorption occurred on the surface of Fe@FeS@C nanospheres (Figure 4d–f). On the surface of the Fe@FeS@C nanoparticles, new and smaller nanoparticles emerged, and firmly adhered to the Fe@FeS@C nanoparticles. By the measurement of crystal spacing, it was confirmed that the new nanoparticles were monomeric silver (Figure 4f). These results suggested that Ag(I) was adsorbed onto the surface of Fe@FeS@C nanoparticles, and was subsequently reduced to Ag monomer through redox reaction.

The Fe 2p high‐resolution energy spectrum of CFeS aerogel demonstrated a marked elevation in the Fe(III) content and a reduction in Fe(II) content after Ag(I) adsorption (Figure 4g). The ratio of Fe(III) to Fe(II) increased from 1:2.1 to 4.3:1 after Ag(I) adsorption. The S 2d energy spectrum revealed that there was no change of the S 2p peaks (Figure 4h), indicating that S was a nonparticipant during the Ag(I) absorption.^17,40,41^ Furthermore, a significant decrease in the peak area corresponding to Fe‐S was observed, suggesting the consumption of a portion of FeS during the Ag(I) adsorption process. The C 1s high‐resolution energy spectrum showed that there was no change in the position and area ratio of the C 1s peaks before and after the Ag(I) adsorption (Figure S12, Supporting Information), indicating that carbon was not involved in the adsorption and oxidation‐reduction of Ag(I). Therefore, the FeS component in the CFeS aerogel reduced Ag(I) to Ag monomer.

Raman spectra of the CFeS aerogel provided additional evidence of the redox reaction between FeS and Ag(I) (Figure 4i). The absorption peaks of CFeS aerogel at 215 cm^−1^, 276 cm^−1^, and 385 cm^−1^ were attributed to asymmetric and symmetric stretching vibrations of Fe‐S, respectively.^[^ ^39^ ^]^ The peaks at 1330 cm^−1^ and 1590 cm^−1^ were attributed to the in‐plane vibrations of disordered amorphous carbon (D band, I_D_) and crystalline graphitic carbon (G band, I_G_), respectively.^[^ ^40^ ^]^ After Ag(I) adsorption, all three peaks of FeS were significantly weakened, and a new peak emerged at 675 cm^−1^, which was the symmetric stretching vibration of Fe─O in FeOOH.^[^ ^41^ ^]^ These results demonstrated that the transition from Fe(II) to Fe(III) occurred during Ag(I) adsorption. In addition, the value of I_D_/I_G_ increased slightly, indicating that the disorder degree of the carbon surface increased after Ag(I) adsorption.^[^ ^42^, ^43^ ^]^ Thus, Ag(I) was reduced by FeS on the carbon surface.

The redox potential of Fe(II) to Fe(III) is 0.77 V, which is positioned above those of Ag(I)→Ag(0) (0.80 V), Pd(II)→Pd(0) (0.92 V) and AuCl_4_^−^→Au(0) (1.00 V), and is below those of other metal ions→zero‐valent metals (Figure 4j). Therefore, FeS can reduce only Ag(I), Pd(II), and Au(III). Since the reduction of Ag(I), Pd(II) and Au(III) requires one, two and three electrons, respectively, the theoretical adsorption amount ratio of Ag(I), Pd(II) and Au(III) is 6:3:2. The actual ratio of the adsorption amount of Ag(I) to Pd(II) was close to 2:1. The big potential difference between Au(III)→Au(0) and Fe(II)→Fe(III) facilitated more FeS participating in the redox process, thereby enhancing the adsorption amount of Au(III). The adsorption kinetic experiments of the CFeS aerogel for Au(III)‐Ag(I)‐Pd(II) mixed solutions (Figure S13, Supporting Information) also demonstrated that, under coexisting conditions of the three ions, the adsorption rate sequence of the CFeS aerogel for the metal ions (Au(III) > Pd(II) > Ag(I)) exhibits exact correspondence with the order of potential differences (ΔE = E(M^n+^/M°) – E(Fe(II)/Fe(III)): ΔE_Au_ = 0.23 V > ΔE_Pd_ = 0.15 V > ΔE_Ag_ = 0.03 V). These data collectively indicate that the unique redox potential of FeS is one of the primary reasons for the exceptional adsorption selectivity of the CFeS aerogel. Although the redox potential could well explain the selective reduction of Ag(I), Pd(II), and Au(III) by FeS, it could not entirely elucidate the ultra‐high selectivity of CFeS aerogel. As demonstrated in Figure S14 (Supporting Information), FeS exhibits equally high adsorption capacities for metal ions including Cu(II), Pb(II), and Cd(II), which can be attributed to the solubility product‐controlled ion exchange reactions between these metals and FeS.^[^ ^44^, ^45^ ^]^ We propose that the 2‐nm‐thick carbon layer encapsulating FeS plays a critical role in suppressing undesired ion‐exchange reactions by preventing metal ion penetration into the core via its compact structure. Experimental evidence strongly supports this mechanism: As shown in Figure S15 (Supporting Information), after adsorption in a mixed solution containing high concentrations (1000 ppm) of Ag(I), Pb(II), and Co(II), no Co or Pb elements were detected in the Fe@FeS@C core‐shell structure, while Ag(I) was exclusively enriched on the outer carbon surface (Figure. 4d–f and S15f, Supporting Information). Furthermore, the highly ordered sp^2^‐hybridized carbon framework (Figure 4i) exhibits superior carrier mobility, resulting in an 80% enhancement in bulk conductivity for CFeS compared to uncoated FeS (Figure S16, Supporting Information). This structural feature significantly facilitates electron transfer from Fe(II) to Ag(I), thereby optimizing the redox selectivity. Therefore, CFeS aerogels exhibited ultra‐high ion selectivity under the synergistic effect of the FeS intermediate layer with a distinctive redox potential and the carbon outer layer with the barrier effect and electron conduction property.

Except the effect to improve selectivity, the carbon layer contributed to enhance the adsorption capacity also. Before and after Ag(I) adsorption, the molar change of S, Fe and Ag in CFeS aerogel was not 1:1:1, but 1:1.51:2.33, signifying that 1 mol Fe(II) ion could effectively reduce 1.5 mol Ag(I) (Figure S17, Supporting Information). The dense carbon layer ensured that Fe(III) ion, which formed after the reaction FeS + Ag^+^ = Fe^3+^ + Ag^0^ +S^2−^, did not diffuse to the external solution immediately. Instead, Fe(III) ion partially reacted with the Fe^0^ in the core to replenish the consumed Fe(II) (via the reaction Fe + 2Fe^3+^ = 3Fe^2+^), making the increase of reduced Ag amount.

Therefore, the mechanism of high selective adsorption of CFeS aerogel for precious metals was as follows (Figure 4k). CFeS aerogel has a distinctive string‐bead network structure, including carbon nanofibers and Fe@FeS@C nanoparticles. Fe@FeS@C nanoparticles were immobilized on carbon nanofibers. The unique redox potential of FeS endowed CFeS aerogel with a reduction selectivity for Ag(I), Pd(II), and Au(III), meanwhile, the carbon layer prevented the ions diffusion into the interior of Fe@FeS@C nanoparticles. The Fe^0^ core provided the magnetic separability, meanwhile, could replenish the depleted Fe(II) to enhance the adsorption capacity. The special string‐bead nanostructure avoided the aggregation and shedding of previous metals during the adsorption process, and expanded the contact area, thus accelerating the adsorption rate and improving the adsorption amount. As a result, CFeS aerogel offered superior selectivity, high efficiency, and easy separation for the enrichment and separation of Ag(I), Pd(II) and Au(III).

### 2.4 Recycling Precious Metals From Wastewater

**Figure 5:**
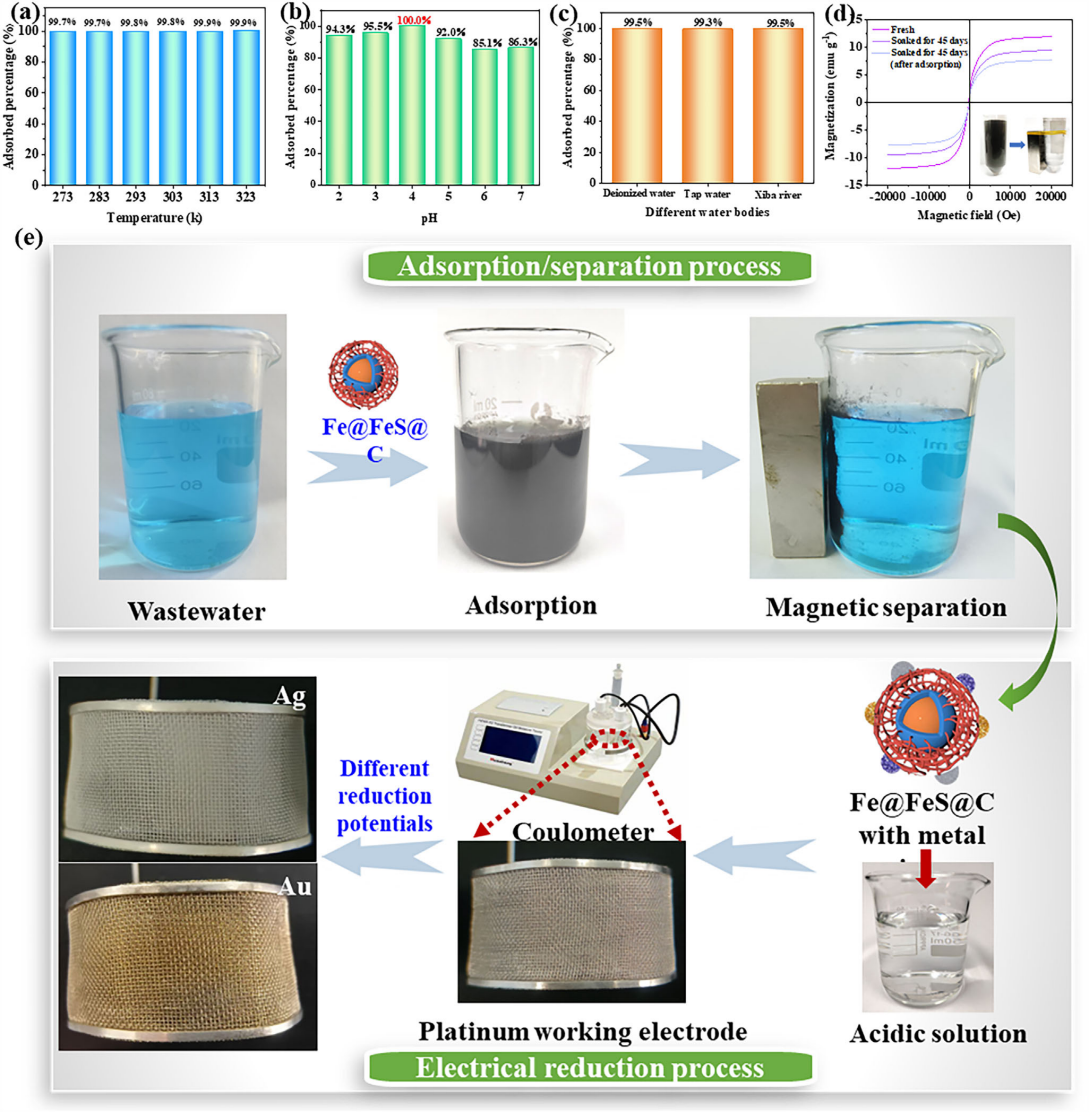
Recycling of Ag and Au by using the CFeS aerogel. Adsorption percentage of Ag(I) by CFeS aerogel a) at different temperatures (273–323 K), b) at different pH (2–7), and c) in different water bodies. d) Magnetic properties of fresh, soaked in water for 45 days, and used CFeS aerogels. e) Recovery process of Ag(I) and Au(III) from wastewater containing 7 kinds of common interfering ions.

We used CFeS aerogels successfully recovered Ag(I) from aqueous bodies with different temperatures, pH and sources (**Figure** **5**). The equilibrium adsorption percentage of Ag(I) by CFeS aerogel exceeded 99% over the temperature range of 273–323 K, demonstrating a high stability (Figure 5a). Thermodynamic parameters including enthalpy change (ΔH_0_), entropy change (ΔS_0_), and Gibbs free energy change (ΔG_0_) of this adsorption process were calculated (Table S3; Figure S18, Supporting Information). A positive ΔH_0_ revealed that the endothermic nature of the adsorption process of Ag(I) by CFeS aerogel.^[^ ^46^ ^]^ A positive ΔS_0_ suggested that as the adsorption process proceeded, the interface between Ag(I) and adsorbent became more disordered. A negative ΔG_0_ confirmed that the adsorption process was spontaneous. Moreover, as the temperature increased, the ΔG_0_ became more negative, underscoring the thermodynamic preference for the Ag(I) adsorption at an elevated temperature.

The adsorption percentage of Ag(I) by CFeS aerogel was notably enhanced under acidic conditions when compared with neutral conditions (Figure 5b). Given that industrial wastewater typically exhibits acidity, CFeS aerogel was suitable to the real‐world applications. Meanwhile, the adsorption percentage showed a trend of initial increase followed by a decrease with the increase of pH values. At pH = 4, the adsorption percentage of Ag(I) by CFeS aerogel was the maximum. This variation was likely attributed to the charged state of the adsorbent surface and the form of metal ions present at different pH. The CFeS aerogel exhibited different charged states in solution with the change of pH (Figure S19, Supporting Information). As the pH increased, the surface charge transitioned from positive to negative. The isoelectric point achieved when the pH was 5. In addition, the reduction of Ag(I) was accomplished by the electron donation from FeS, thus the CFeS aerogel with a positively charged surface promoted electron transfer from FeS to the outer carbon layer. However, when the CFeS aerogel surface became excessively electropositive, the electrostatic repulsion would impede the approach of Ag(I) ions. Therefore, the optimal adsorption was achieved at a balance between the electron transfer and the electrostatic repulsion on the surface of the CFeS aerogel. Additionally, Ag(I) predominantly existed as Ag(OH) in near‐neutral environments, which accounted for the slight reduction in the adsorption percentage at pH > 5. Thus, acidic conditions favored the adsorption of Ag(I) onto the CFeS aerogel. The adsorption percentage of Ag(I) by CFeS aerogel achieved a maximum of 100% at pH = 4.

The real‐world water samples contain not only various metal ions, but also a myriad of other substances that may potentially interfere with the adsorption process. So, a comparative analysis of the adsorption capacity of CFeS aerogel for Ag(I) ion was conducted across different water sources, including distilled water, tap water containing anions, and Xiba River water containing complex substances (Figure 5c). The CFeS aerogel demonstrated an impressive adsorption percentage of over 99% Ag(I) in all three water bodies, which significantly validated its effectiveness in capturing precious metal ions in real‐world aquatic environments. Additionally, after 90 days of storage in ambient air, the adsorption selectivity of the CFeS aerogel remained unchanged, while its adsorption capacities for Ag(I), Pd(II), and Au(III) showed only a slight decrease (<20%) (Figure S20, Supporting Information). Based on XRD analysis (Figure S21, Supporting Information), we propose that this phenomenon arises from the partial oxidation of Fe or FeS in the CFeS aerogel into Fe_2_O_3_, leading to a weakening of its reducing capability. These results indicate that the long‐term stability of CFeS aerogel is excellent.

The separation and recovery of adsorbents are crucial in practical applications. The saturated magnetization strength of CFeS aerogel at room temperature was ≈12 emu·g^−1^ (Figure 5d). This strong ferromagnetic property enabled a rapid magnetic separation of CFeS aerogel, thereby significantly reducing the recovery cost. It was worth mentioning that the CFeS aerogel retained 80% of its initial magnetic property after being soaked in water for 45 days. Even if it underwent an adsorption process under acidic conditions, the soaked CFeS aerogel still retained 65% of its initial magnetic property. The loss of magnetic performance originated from the consumption of Fe^0^ during the adsorption process. But, compared with bare Fe^0^ and Fe_3_O_4_, the CFeS aerogel demonstrated superior resistance to acid and oxidant, thanks to the dual protection of FeS and carbon layer.

To further assess the practical application potential of this material, we evaluated its production cost. As shown in Table S4 and S5 (Supporting Information), the production cost for kilogram‐scale material is ≈$1.3/g. Compared to selective adsorption materials such as covalent organic frameworks, metal‐organic frameworks, and porous organic polymers, the CFeS aerogel demonstrates unique advantages: First, the material can be mass‐produced via mature sol‐gel processes, with its unit cost potentially reduced to below $1/g through optimized production. Second, its high selectivity significantly streamlines subsequent desorption and purification steps, thereby lowering energy consumption in electrolytic reduction (accounts for ≈50% of total precious metal recovery costs). Based on current market prices (Au: $97.6/g, Pd: $31.3/g), the CFeS aerogel offers substantial economic benefits for industrial‐scale precious metal recovery.

Finally, based on the types and concentrations of metal ions reported in the literature for electronic wastewater, gold ore leaching solutions, and electroplating wastewater (Table S6, Supporting Information), we prepared a more challenging mixed solution (with more types of interfering ions and higher concentrations) to validate the practical industrial application potential of our material. As shown in Figure 5e, we conducted a complete recovery process for Au(III) and Ag(I) in simulated wastewater containing seven high‐concentration interfering ions: Cu(II), Al(III), Mn(II), Co(II), Ni(II), Zn(II), and Cd(II). CFeS aerogel was added into the wastewater, stirred for 10 min to complete the adsorption, and separated by using an applied magnetic field. Subsequently, the eluent was used to treat the recycled CFeS aerogel to detach the absorbed metal, and the obtained solution was electrochemically reduced to deposit Ag and Au, respectively (Figures S22 and S23, Supporting Information). This whole recovery process was designed to be completed within 1 h, effectively separating and purifying trace precious metals from complex water bodies. In addition, the CFeS aerogel with the selective adsorption of precious metals holds the potential in the smelting and purification of precious metals. Via simply burning off the carbon in the CFeS aerogel after absorption, metal powders with high‐content precious metals can be obtained.

## 3 Conclusion 

We demonstrated a magnetic carbon aerogel to realize an ultra‐highly selective adsorption of precious metal ions by an interfacial selective‐reduction process. Carbon aerogels embedded with magnetic trilayer Fe@FeS@C nanoparticles (CFeS aerogels) were fabricated by the Fe^3+^‐induced gelation and subsequent carbonization of natural carrageenan bearing sulfonate groups. CFeS aerogels had ultra‐high ion selectivity, and could selectively adsorb and reduce trace Au(III), Ag(I), and Pd(II) ions at ppb level in an aqueous solution with 29 coexisting cations. Even if the concentration of coexisting metal ions was 100 times higher than those of noble metal ions, the selectivity of the CFeS aerogels for Au(III), Ag(I), and Pd(II) ions remained above 99.2%. Such exceptional ion selectivity originated from the FeS intermediate layer with a distinctive redox potential and the carbon outer layer with the barrier effect and electron conduction property. In addition, the 3D carbon network avoided the aggregation and detachment of the precious metal particles during the extraction process, thereby enhancing both the adsorption rate and adsorption capacity. CFeS aerogels demonstrated rapid adsorption kinetics for Au(III), Ag(I) and Pd(II) ions, with saturated adsorption amount of 321.2, 150.6, and 70.1 mg·g^−1^, respectively. Moreover, the inclusion of Fe^0^ endowed the CFeS aerogels with a magnetic separability. CFeS aerogels successfully achieved a selective and rapid recovery of Au and Ag from the simulated industrial wastewater. Thus, CFeS aerogels with a unique selectivity mechanism stand out as an efficient and highly‐selective adsorbent for precious metals, offering a novel insight for the selective adsorption and enrichment of metal ions.

## 4 Experimental section

### Materials

I‐Carrageenan and ferric chloride (FeCl_3_) were obtained from Shanghai Aladdin Biochemical Technology Co., Ltd. The standard solution of metal ions was obtained from the National Institute of Metrology of China. Nitric acid, hydrochloric acid, sodium hydroxide, and ethanol were purchased from Beijing Chemical Reagent Company. All chemicals were analytical pure, and were used directly unless otherwise specified.

### Fabrication of CFeS Aerogel

First, carrageenan solution (3 wt.%) was added drop by drop into FeCl_3_/ethanol solution (1.6 wt%). Under continuous stirring for 45 min at ambient temperature, Fe‐carrageenan hydrogel was obtained. The hydrogel was thoroughly washed with distilled water, and was subsequently placed in a vacuum freeze dryer to freeze‐drying. The resulting Fe‐carrageenan aerogel was transferred to a tube furnace, ramping up to 800 °C at a rate of 2 °C min^−1^ under Ar atmosphere, with a dwell time of 2 h. Upon cooling to room temperature, the carbon aerogel with Fe@FeS@C nanoparticles was obtained.

### Selective Adsorption Experiment

A mixture solution of 50 mL was prepared, containing Mg(II), Al(III), Ca(II), V(V), Cr(VI), Mn(II), Co(II), Ni(II), Cu(II), Zn(II), Ga(III), As(V), Rb(I), Sr(II), Ag(I), Cd(II), In(III), Cs(I), Ba(II), Tl(I), Pb(II), Bi(III), Ru(VI), Rh(III), Pd(II), Sn(II), Sb(V), Ir(III), and Au(III) (100 ppb per metal ion). The CFeS aerogel (10 mg) was added into the above solution. The mixture was continuously stirred for 10 min. An applied magnetic field was used to separate the CFeS aerogel and the solution. Then, the adsorbed solution was filtered through a 0.22 µm filter membrane. The concentration of the metal ions in the above filtrate was determined. The adsorption percentage of the CFeS aerogel for metal ions was calculated by Equation S1 (Supporting Information), the affinity coefficient of the CFeS aerogel for the metal ions was calculated by Equation S2 (Supporting Information), and the selectivity coefficient of the CFeS aerogel for the noble metal ions (Au(III), Ag(I), Pd(II)) was calculated by Equation S3 (Supporting Information).

A mixture solution (50 mL) was prepared, including Co(II), Ni(II), Cu(II), Zn(II), Cd(II), and Pb(II) at concentrations of 10 ppm, and Au(III), Ag(I), and Pd(II) at 100 ppb. The CFeS aerogel (10 mg) was added into the above solution, and the mixture was continuously stirred for 10 min. An applied magnetic field was used to separate the CFeS aerogel and the solution, and then the adsorbed solution was filtered through a 0.22 µm filter membrane. Keeping other conditions unchanged, humic acid (final concentration of 200 ppm) and mixed salts (1 wt% K+/Na+) were separately added to the mixed solution to simulate a more challenging application environment. The concentration of the metal ions in the above filtrate was determined, and the adsorption percentage of the CFeS aerogel for metal ions was calculated by the Equation S1 (Supporting Information).

### Adsorption Kinetics Experiment

Ag(I) solutions with the initial concentrations of 10 ppm and 100 ppb (50 mL) were configured by diluting Ag(I) standard solution with water, respectively. To each solution, the CFeS aerogel (10 mg) was added, and the mixtures were stirred continuously at room temperature. At regular intervals, 5 mL solution was removed from the mixture and was filtered through a 0.22 µm filter membrane. The residual Ag(I) concentration in the filtrate was measured.

The adsorption kinetic experiments for Au(III), Pd(II), and Au(III)/Pd(II)/Ag(I) mixed solutions were conducted using initial concentrations of 10 ppm Au(III) solution, Pd(II) solution, and Au(III)/Pd(II)/Ag(I) solution, respectively. The experiment steps were consistent with the above procedure. The adsorption kinetic data were fitted with a pseudo‐second‐order kinetic equation (Equation S4, Supporting Information).

### Isothermal Adsorption Experiment

A series of 20 mL Ag(I) solution with different concentrations were configured, and their pH was adjusted to 4 using dilute nitric acid (HNO_3_) and sodium hydroxide (NaOH). To each solution, CFeS aerogel (2 mg) was added, and the mixture was stirred for 15 min to achieve adsorption equilibrium. Then, the solution was filtered through a 0.22 µm filter membrane, and the concentration of Ag(I) in the filtrate was determined. The equilibrium adsorption capacity (Q_e_, mg·g^−1^) of CFeS aerogel for Ag(I) was calculated by Equation S5 (Supporting Information). The isothermal adsorption experimental data were fitted with the Langmuir isothermal adsorption model (Equation S6, Supporting Information) and the Freundlich isothermal adsorption model (Equation S7, Supporting Information), respectively.

The isothermal adsorption experimental procedures for Au(III) and Pd(II) were the same as described above, except that the Ag(I) solution was replaced with Au(III) and Pd(II) solution, respectively.

### Adsorption Thermodynamic Experiment

Ag(I) solution (1 ppm) was treated with CFeS aerogel (2 mg) at different temperatures (0, 10, 20, 30, 40, and 50 °C). The feasibility and spontaneity of Ag(I) adsorption by CFeS aerogel were assessed by the estimation of enthalpy change (ΔH_0_), entropy change (ΔS_0_), and free energy change (ΔG_0_). The thermodynamic parameters were calculated by Equations S8–S10 (Supporting Information).

### Effect of pH on Adsorption

The pH values of aqueous solutions of Ag(I) (10 ppm) were adjusted to 2, 3, 4, 5, 6, and 7, respectively. Then, an equal amount of CFeS aerogel was added, and the mixture was stirred continuously for 10 min. Finally, the solution was filtered through a 0.22 µm filter membrane. The residual concentration of Ag(I) in the filtrate was measured, and the adsorption percentage was calculated.

### Recovery of Au and Ag From Wastewater

Prepared a simulated industrial wastewater solution with a Cu(II) concentration of 100 ppm, and Al(III), Mn(II), Co(II), Ni(II), Zn(II), Cd(II), Pb(II), Ag(I), and Au(III) concentrations of 10 ppm each. CFeS aerogel was added into the solution, stirred for 10 min, and then magnetically separated. Subsequently, the CFeS aerogel was immersed in aqua regia, and the immersion solution was reduced by a three‐electrode setup (platinum mesh as the working electrode, platinum wire as the counter electrode, and saturated calomel electrode as the reference electrode). The reduction potentials were specifically set at 0.20 V for Ag and 0.45 V for Au.

### Characterization

X‐ray diffraction (XRD) patterns were recorded on an X‐ray diffractometer (Bruker, D8 Focus). Scanning electron microscopy (SEM) observation was carried out on a field‐emission scanning electron microscope (ZEISS, Gemini 500). High‐resolution transmission electron microscopy (HRTEM) observation and electron dispersive spectroscopy (EDS) measurement were performed on a transmission electron microscope (JEOL, JEM‐2100). X‐ray photoelectron spectroscopy (XPS) test was carried out on a XPS spectrometer (Thermo Fisher Scientific, ESCALAB 250Xi), and all data were calibrated by using the binding energy of the C 1s peak at 284.8 eV. The pH values were measured by using a Five Easy Plus pH meter (Mettler Toledo, FE28). The concentrations of metal ions were detected by using HR‐ICP‐MS (Thermo Fisher Scientific ELEMENT 2). Brunauer‐Emmett‐Teller (BET) specific surface areas were obtained on an automatic specific surface area and pore size analyzer (Quantachrome, Quadrasorb SI‐MP). Zeta potential of the solution at different pH was tested by a Nano particle sizer (Malvern, Zetasizer Nano ZS90). Magnetization measurements were performed at 295 K on a magnetic measurement system (Quantum Design company MPMS‐3). Raman spectra were obtained on a Via‐Reflex Renishaw raman spectrometer (Renishaw, inVia‐Qontor) with an incident wavelength of 532 nm. Fourier‐transform infrared (FTIR) spectra were recorded in a range of 300–4000 cm^−1^ at a resolution of 2 cm^−1^ on a Varian Excalibur 3100 spectrometer. Carbon and sulfur content was tested by carbon‐sulfur meter (Leco, CS844‐MC). The electrical conductivity of the material was measured using a four‐point probe meter (Guangzhou Four‐Probe Technology Co., Ltd., RTS‐8). Electric reduction experiments were accomplished by a coulometer (Beijing Tongzhou Weipu Science and Technology Co., Ltd., CP‐15001), with platinum as the working and counter electrodes and a saturated mercuric glycol electrode as the reference electrode.

## Conflict of interest

The authors declare no conflict of interest.

## Supporting information



Supporting Information

## Data Availability

The data that support the findings of this study are available in the supplementary material of this article.
