# Supplementary material for: Superior‐Selective and Complete Recycling of Trace Precious Metals From Wastewater by Magnetic Trilayer Carbon‐Aerogels
Source: Adv Sci (Weinh). 2025 May 28;12(31):e00858. doi: 10.1002/advs.202500858 (PMC12376680; doi:10.1002/advs.202500858)
Supplement: Supplementary file 1 — Supporting Information [file ADVS-12-e00858-s001.pdf]

ADVANCED SCIENCE
Open Access

Supporting Information

for *Adv. Sci.*, DOI 10.1002/advs.202500858

Superior-Selective and Complete Recycling of Trace Precious Metals From Wastewater by Magnetic Trilayer Carbon-Aerogels

Jianzheng Yang, Yan Zhou, Shang Du, Bing Wu, Jianying Zhang, Shanjun Song, Tao Zhou and Jinming Zhang**

Supplementary Information

Superior-Selective and Complete Recycling of Trace Precious Metals from Wastewater by Magnetic Trilayer Carbon-Aerogels

Jianzheng Yang[a], Yan Zhou[a], Shang Du[a], Bing Wu[a], Jianying Zhang[a], Shanjun Song[a], Tao Zhou[a,*], Jinming Zhang[b,*]

a National Institute of Metrology, Beijing 100013, China
b CAS Key Laboratory of Engineering Plastics, Institute of Chemistry, Chinese Academy of Sciences (CAS), Beijing 100190, China
* Corresponding author. E-mail address: zhoutao@nim.ac.cn; zhjm@iccas.ac.cn

Supporting information content

Number of pages: 23
Number of figures: 23
Number of tables: 6
Number of equations: 10

Calculation of metal ion adsorption percentage

The removal percentage of metal ion in the solution is calculated by the formula:

$$\text{Removal (\%)} = \frac{C_0\text{-}C_t}{C_0} \times 100\% \tag{S1}$$

where C_0 and C_t represent the metal ion concentrations at the initial and time t, respectively.

Calculation of distribution coefficient and selectivity factor

The distribution coefficient K_d is a parameter to evaluate the affinity between the adsorbent and the adsorbate, which can be determined by

$$k_d = \frac{C_0\text{-}C_e}{C_e} \times \frac{V}{m} \tag{S2}$$

where C_0 and C_e are the initial concentration and the equilibrium concentration of metal ion, respectively. V is the solution volume, and m is the mass of CFeS aerogel used.

The selectivity factor $\alpha_{TM/OM}$ is used to assess the ability of the adsorbent to differentiate between target metal ions and other metal ions, which can be calculated as:

$$\alpha_{TM/OM} = \frac{K_{d(TM)}}{K_{d(OM)}} \tag{S3}$$

where $K_{d(TM)}$ and $K_{d(OM)}$ are the distribution coefficients of the target metal ions and other metal ions respectively.

Adsorption kinetics model

Pseudo second-order kinetic equation:

$$\frac{t}{Q_t} = \frac{1}{k_2 Q_e^2} + \frac{t}{Q_e} \tag{S4}$$

where k_2 (g mg^{-1} min^{-1}) is the pseudo-second-order rate constant of adsorption, Q_t and Q_e represent the amounts of metal ion adsorbed by CFeS aerogel at time t and at equilibrium, respectively.

Calculation of equilibrium adsorption capacity

$$Q_e = \frac{(C_i - C_e)}{m} \times V \quad \text{(S5)}$$

where C_e denotes to the equilibrium concentration of metal ions, V (mL) is the solution volume, and m (g) is the amount of utilized adsorbent.

Equilibrium models

Langmuir isotherm model

The Langmuir isotherm model can display the monolayer molecule adsorption onto homogeneous active sites of adsorbents. The Langmuir model is expressed linearly as:

$$\frac{C_e}{Q_e} = C_e \frac{1}{Q_{max}} + \frac{1}{K_L Q_{max}} \quad \text{(S6)}$$

where Q_{max} (the maximum adsorption capacity, mg g^{-1}) and K_L (the Langmuir constant, L mg^{-1}) can be obtained by plotting C_e/Q_e *versus* C_e. C_e and Q_e are the concentration and adsorption amount at equilibrium, respectively.

Freundlich isotherm model

The Freundlich isotherm model depicts the homogeneous active sites on the adsorbent surface and is always used to describe the multilayer adsorption. The Freundlich model is expressed linearly as:

$$LnQ_e = LnK_F + \frac{1}{n} LnC_e \quad \text{(S7)}$$

where K_F (the Langmuir constant, L mg^{-1}) and n can be obtained by plotting LnQ_e *versus* LnC_e. The 1/n represents the Freundlich adsorption constant related with adsorption intensity.

Calculation of thermodynamic parameter

Three important thermodynamic parameters such as Gibbs free energy change (ΔG_0, kJ mol^{-1}), adsorptive enthalpy change (ΔH_0, kJ mol^{-1}) and adsorptive entropy change (ΔS_0, J mol^{-1} K^{-1}) are calculated from the following equations:

$$K_d = \frac{Q_e}{C_e} \quad \text{(S8)}$$

$$\triangle G_0 = -RT \ln K_d \quad \text{(S9)}$$

$$\ln K_d = \frac{\triangle S_0}{R} - \frac{\triangle H_0}{RT} \tag{S10}$$

where T is the temperature, R is the gas constant, 8.314 J mol^{-1} K^{-1}.

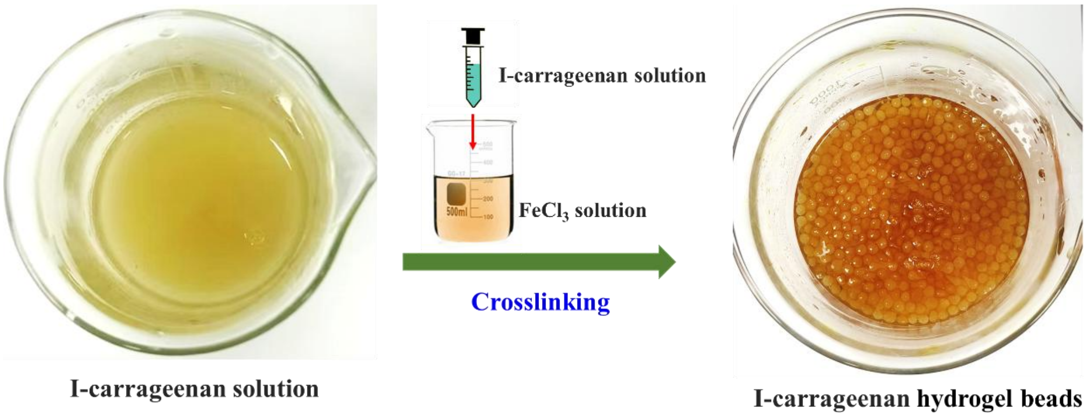

Fig. S1 Optical photos of the formation of carrageenan-Fe(III) hydrogel spheres.

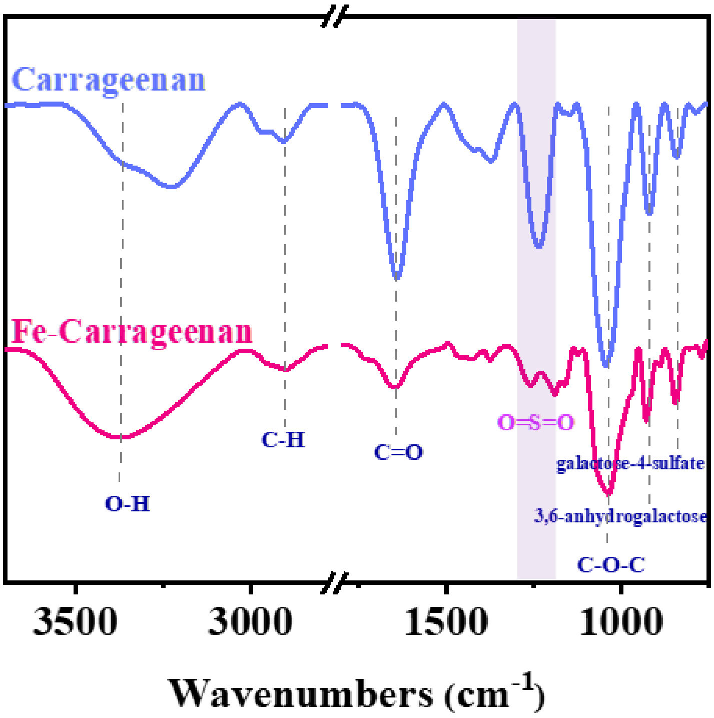

Fig. S2 FTIR spectra of carrageenan aerogel and carrageenan-Fe(III) aerogel.

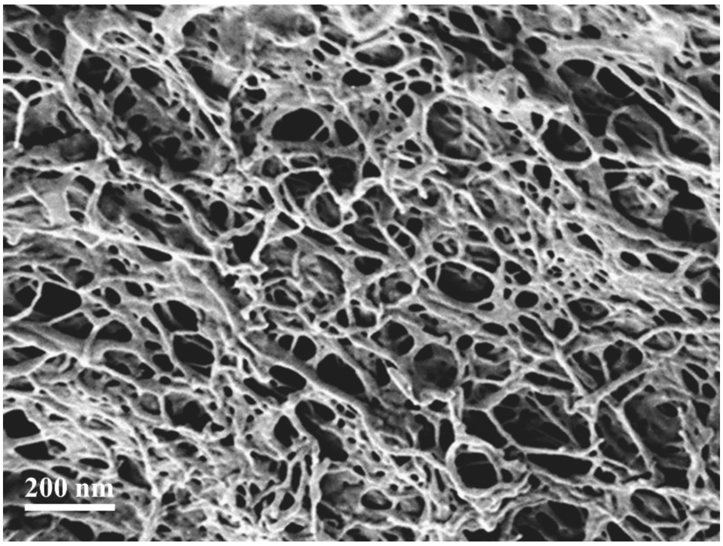

Fig. S3 SEM image of carrageenan-Fe(III) aerogel.

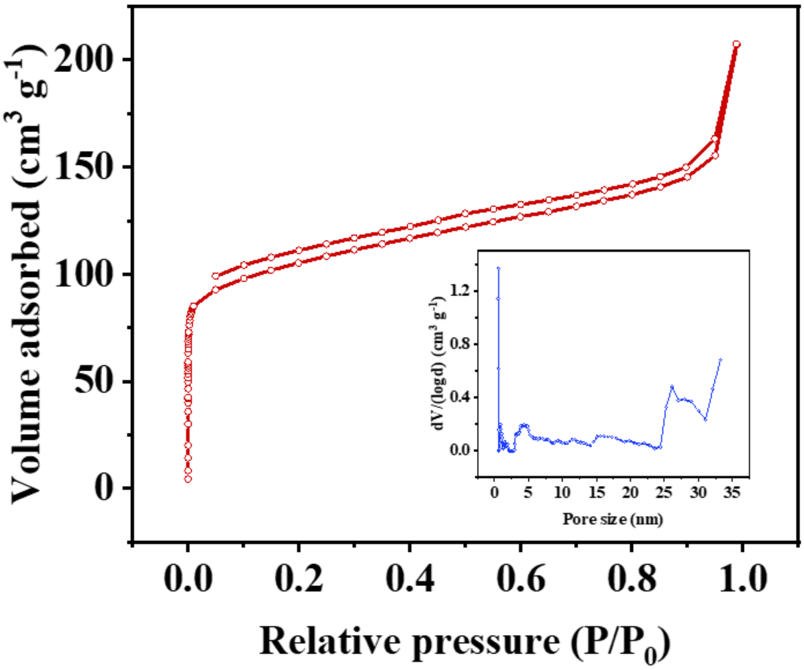

Fig. S4 N_2 adsorption/desorption isotherms of CFeS aerogel. The inset figure is the pore size distribution.

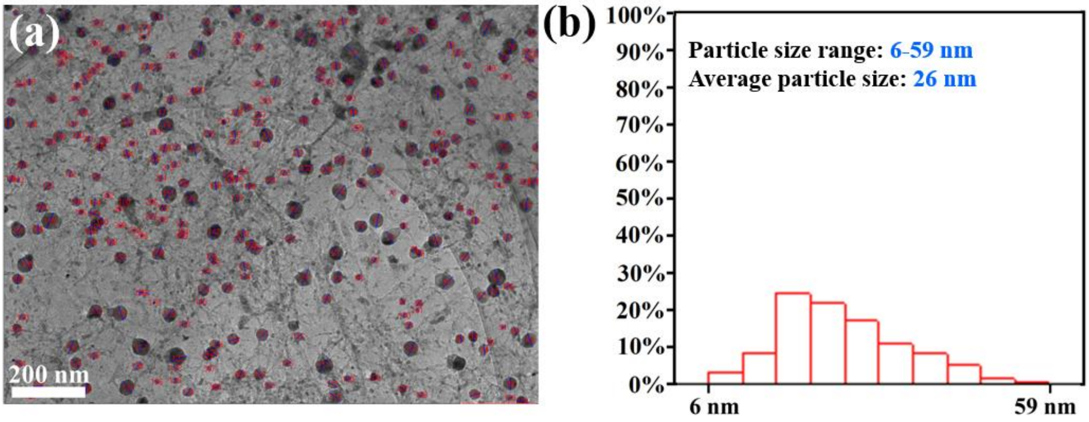

Fig. S5 Particle size statistics of Fe@FeS@C nanoparticle. (a) SEM image for statistical particle size; (b) Particle size distribution.

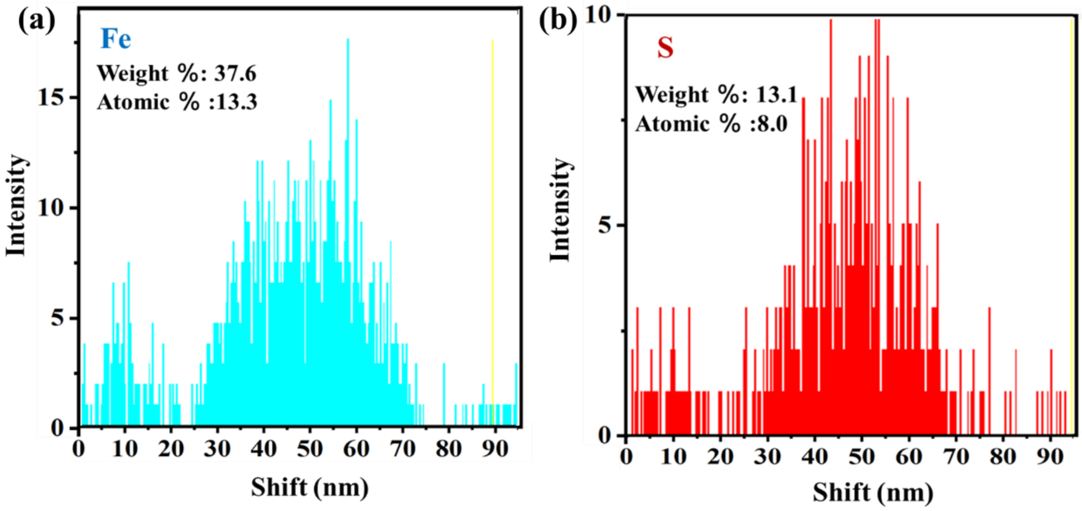

Fig. S6 Distribution of element content in single Fe@FeS@C nanoparticle. (a) Fe; (b) S.

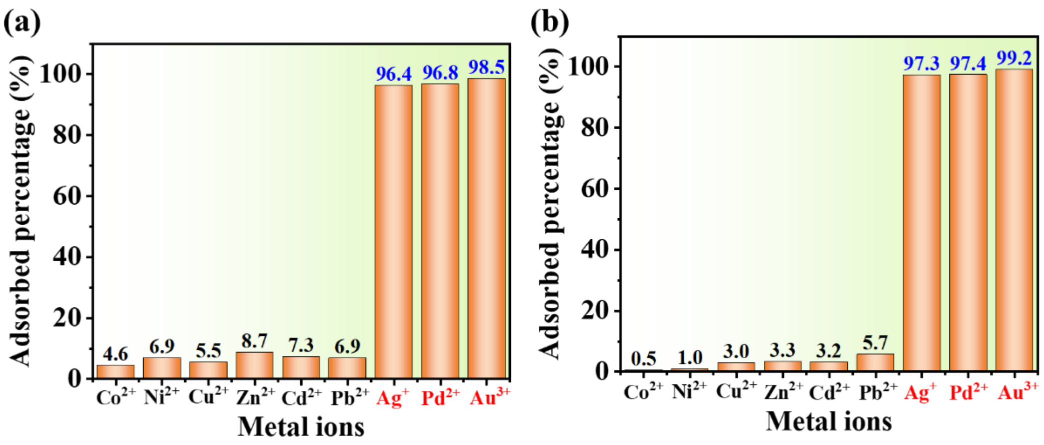

Fig. S7 Selective adsorption performance of CFeS aerogel for trace noble metals in complex aqueous environments. (a) Humic acid coexisting system (200 ppm); (b) High-salinity interference system (1 wt% K^+/Na^+ mixed salts). The test system contains 6 competitive heavy metal ions (Co(II), Ni(II), Cu(II), Zn(II), Cd(II), Pb(II), each at 100 ppm) and 3 noble metal ions (Ag(I), Pd(II), Au(III), each at 100 ppb).

Table S1 Comparison of the adsorption properties of magnetic adsorbents for Au(III), Ag(I) and Pd(II) in aqueous solution.

Adsorbent	Target metal ions	Number of coexisting ions	Interfering ions (>5%)	Contact time	Adsorption capacity
FO@SD@CS [1]	Ag(I)	6	Cu, Pb	200 min	81.60 mg/g
MNSR-DM-AQ [2]	Pd(II)	9	Ag, Al, Co, Sn, Ni, Cu, Zn	30 min	11.9 mg/g
Fe_3O_4@SiO_2-NH_2 nanoparticles [3]	Au(III), Pd(II), Pt(IV)	5	N. A.	30 min	69.71 mg/g for Au(III), 32.38 mg/g for Pd(II), 12.50 mg/g for Pt(IV)
$CoFe_2O_4$@S–	Au(III)	7	Pd, Ca, Al	240 min	1049 mg/g

$CoWO_4$ [4]					
A-2M@Fe_3O_4 [5]	Au(III)	7	Pt, Hg	10 h	464.8 mg/g
B-CPCMFA [6]	Ag(I)	3	Cu, Zn	350 min	57.02 mg/g
EG/MNPs [7]	Pd(II)	6	N.A.	180 min	26.32 mg/g
CFeS aerogel (This work)	Ag(I), Pd(II), Au(III)	26	N.A.	10 min for Ag(I) and Pd(II); 5 min for Au(III)	150.6 mg/g for Ag(I), 70.1 mg/g for Pd(II), 321.2 mg/g for Au(III)

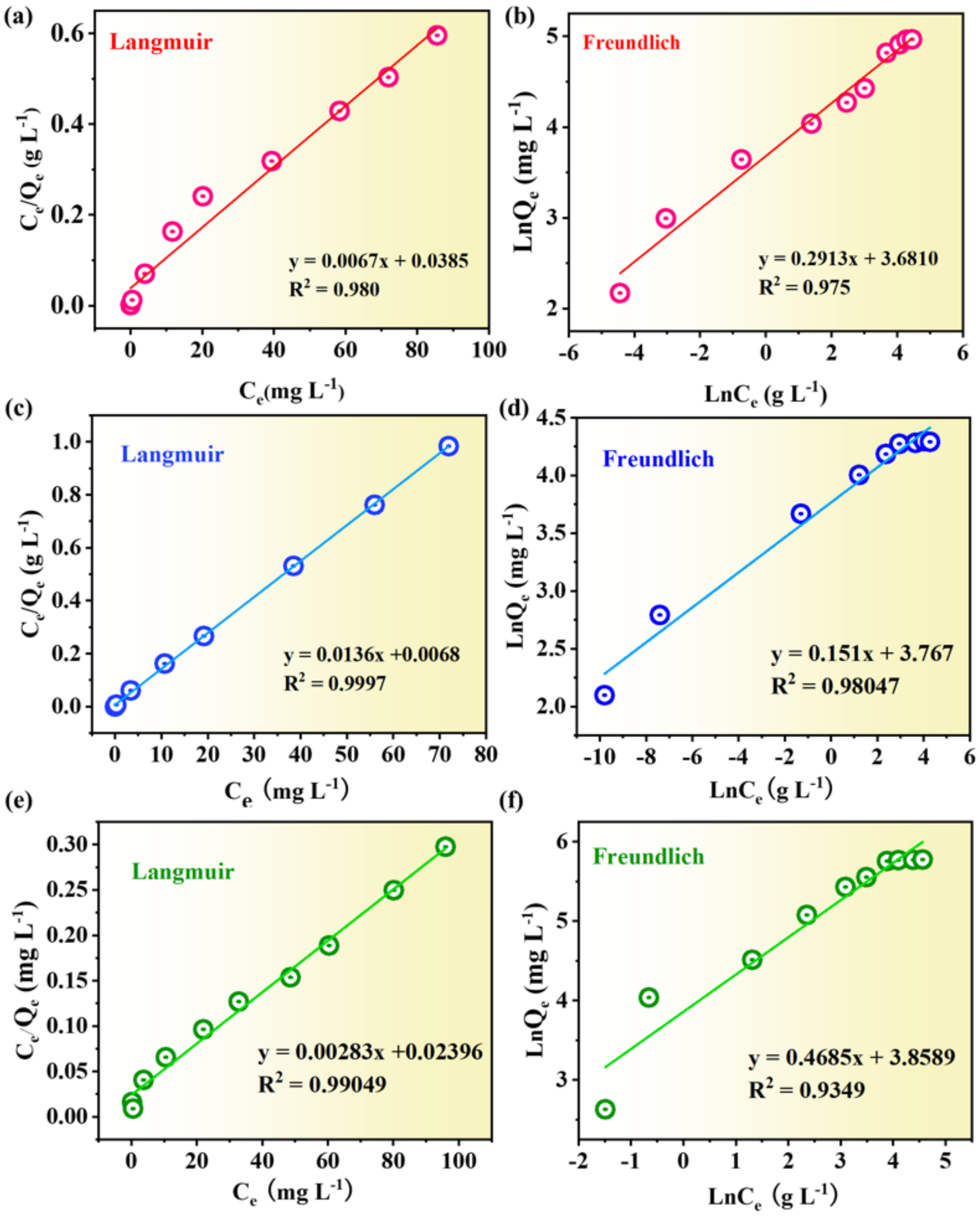

Fig. S8 Fitting result of different metal ions adsorption by isotherm models. Fitting result of Ag(I) adsorption by (a) Langmuir and (b) Freundlich isotherm models. Fitting results of Pd(II) adsorption by (c) Langmuir and (d) Freundlich isotherm models. Fitting results of Au(III) adsorption by (e) Langmuir and (f) Freundlich isotherm models.

Table S2 Langmuir and Freundlich model parameters for different metal ions adsorption over CFeS aerogel.

Metal ion	Model	K (L mg^{-1})	n	Q_{max} (mg g^{-1})	R^2
**Ag(I)**	Langmuir	0.17		149.3	0.980
	Freundlich		3.4		0.975
**Pd(II)**	Langmuir	0.20		73.5	0.997
	Freundlich		6.6		0.980
**Au(III)**	Langmuir	0.118		353.4	0.990
	Freundlich	13.24	2.1		0.935

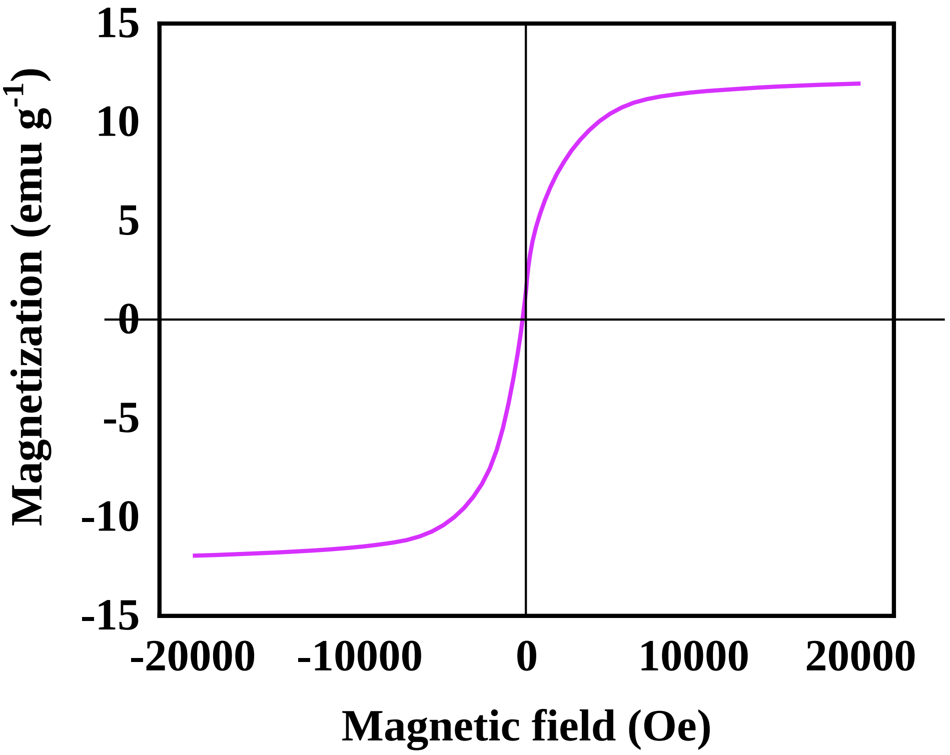

Fig. S9 Magnetic property of CFeS aerogel.

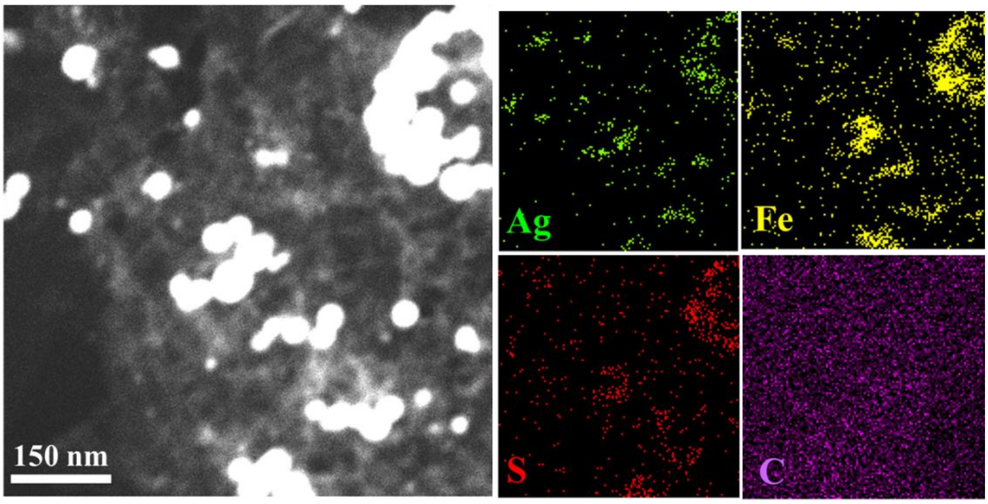

Fig. S10 TEM image and corresponding EDS elemental mapping images of Ag, Fe, S and C in CFeS aerogel after Ag(I) uptake.

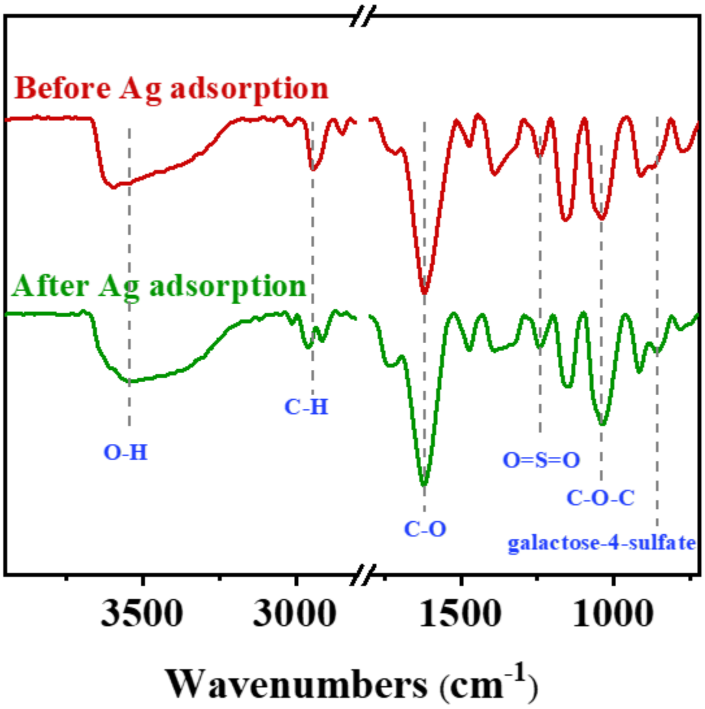

Fig. S11 FTIR spectra of CFeS aerogel before and after adsorption of Ag(I).

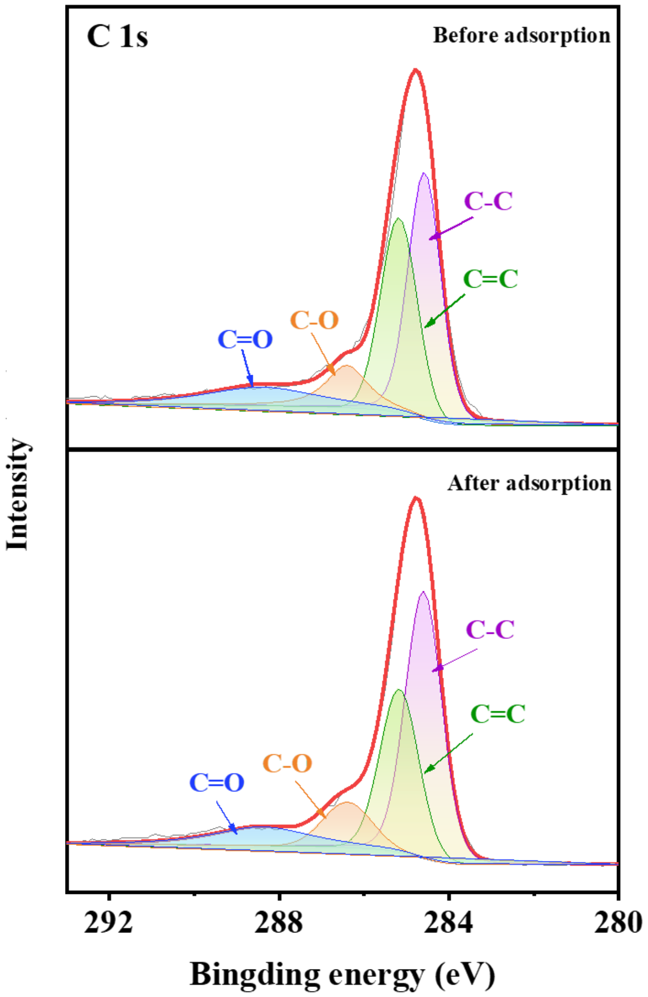

Fig. S12 High-resolution C 1s XPS spectra of CFeS aerogel before and after Ag(I) adsorption.

Metal-Chloride Complexation Analysis

The stability of chloride complexes was evaluated using stability constants (K) from the critical stability constants database. The 10ppm standard solution used for adsorption experiments was prepared by diluting 10000ppm standard solution (with a substrate of 2% (v/v) aqua regia). Consequently, the Cl^- concentration in the experimental solution was 1.8×10^{-4} M.

For Au^{3+} system, due to the strong tendency of Au^{3+} to form stable $AuCl_4^-$ complexes in Cl^--containing solutions ($Au^{3+} + 4Cl^- \rightleftharpoons AuCl_4^-$), the stability constant is defined as:

$$K = \frac{[AuCl_4{}^{-}]}{[Au^{3+}][Cl^{-}]^4} = 10^{26} \text{ (at 25 °C)}$$

The ratio of complexed to free ions becomes:

$$\frac{[AuCl_4{}^{-}]}{[Au^{3+}]} = K[Cl^{-}]^4 \approx 10^{11}$$

This demonstrates that $AuCl_4^-$ overwhelmingly dominates (>99.99%) in solution. Thus, $AuCl_4^-$/Au (1.00 V) is the correct redox pair.

For Pd(II) system, although $PdCl_4^{2-}$ complexes exist, their significantly lower stability constant ($K = 10^{12}$) leads to fundamentally different speciation under identical Cl^- concentrations. The ratio of complexed to free ions becomes:

$$\frac{[PdCl_4{}^{2-}]}{[Pd^{2+}]} = K[Cl^{-}]^4 \approx 10^{-3}$$

This indicates that >99% of palladium exists as free Pd^{2+} ions, validating the use of the Pd^{2+}/Pd (0.92 V) standard redox potential.

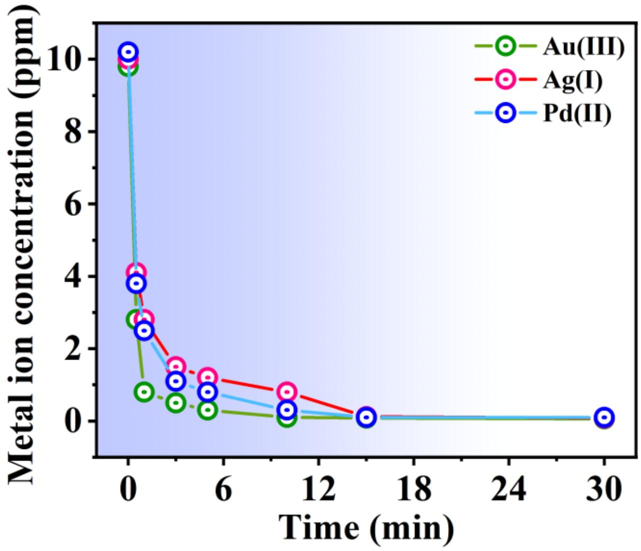

Fig. S13 Adsorption kinetics of the CFeS aerogel for Au(III), Ag(I), and Pd(II) in mixed solutions (Initial concentrations of Au(III), Ag(I), and Pd(II) were all 10 ppm).

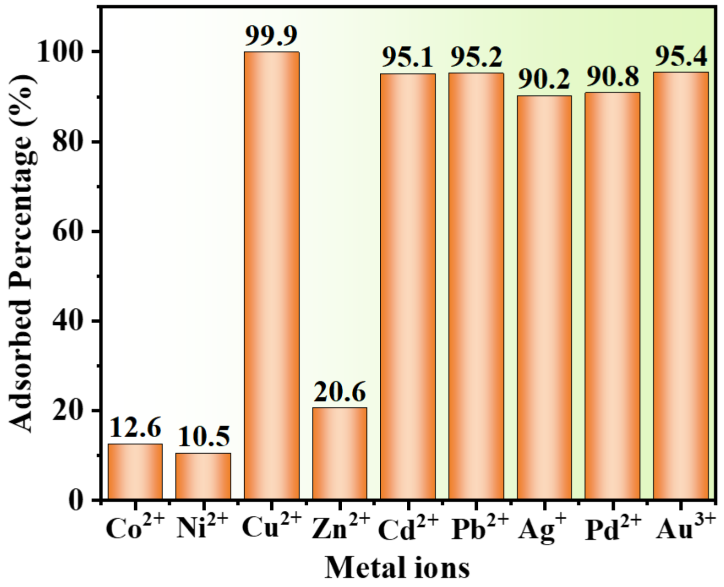

Fig. S14 Adsorption selectivity of FeS among 9 metal ions (100 ppb per ion).

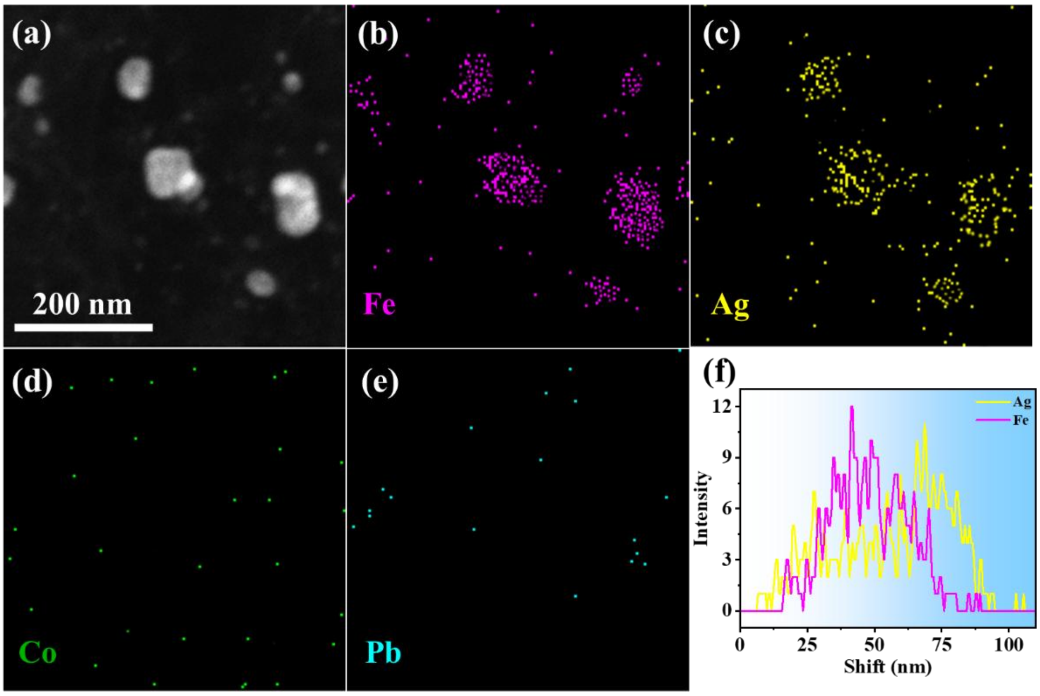

Fig. S15 TEM images and EDS elemental mapping of CFeS aerogel after selective adsorption of metal ions. (a) TEM image; (b) Fe distribution; (c) Ag accumulation; (d) Co exclusion; (e) Pb exclusion; (f) Distribution of Fe and Ag content in single Fe@FeS@C nanoparticle after Ag(I) adsorption.

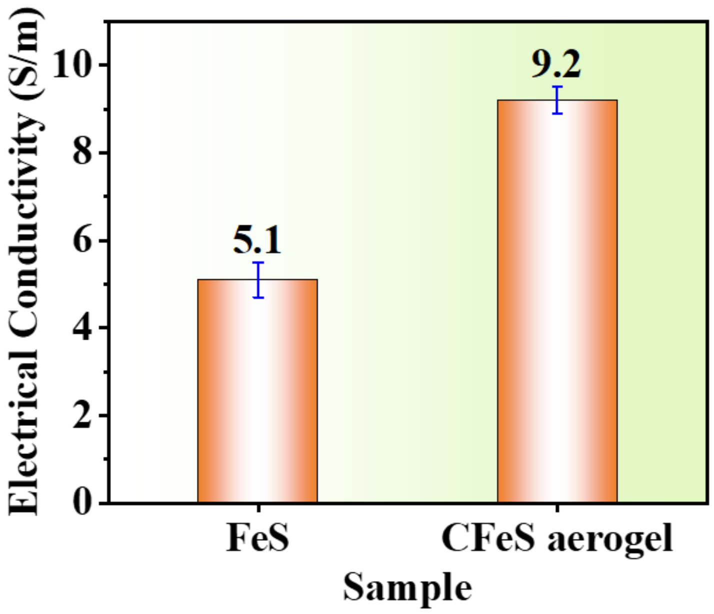

Fig. S16 Electrical conductivity of FeS and CFeS aerogel.

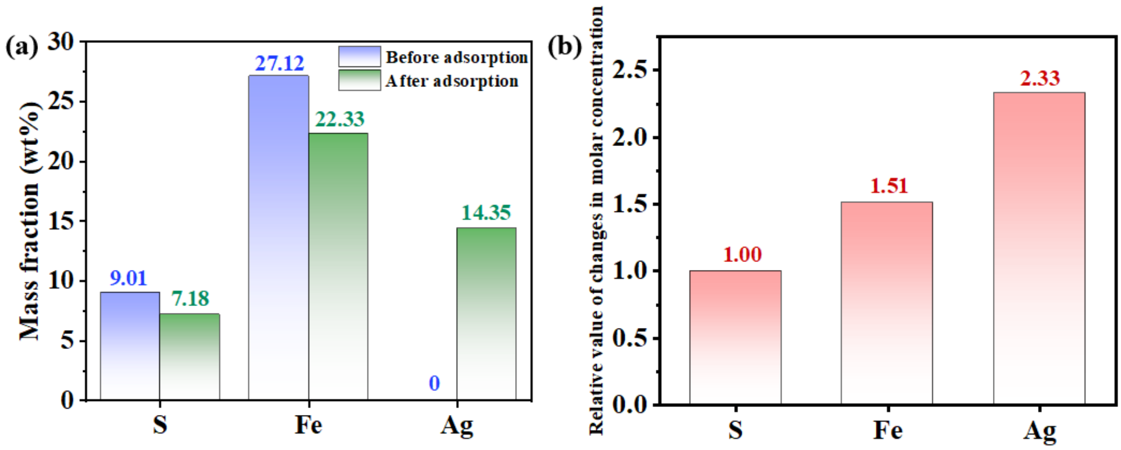

Fig. S17 Elemental concentration analysis of bulk CFeS aerogel before and after Ag(I) adsorption via carbon/sulfur analyzer and ICP-OES. (a) Mass concentration; (b) Molar concentration.

Table S3 Thermodynamic parameters for Ag(I) adsorption on CFeS aerogel.

T (°C)	$\triangle H_0$ (kJ·mol^{-1})	$\triangle S_0$ (J·mol^{-1}·K^{-1})	$\triangle G_0$ (kJ·mol^{-1})
0			-32.18
10			-33.92
20	15.26	173.68	-35.65
30			-37.39
40			-39.13

50	-40.86

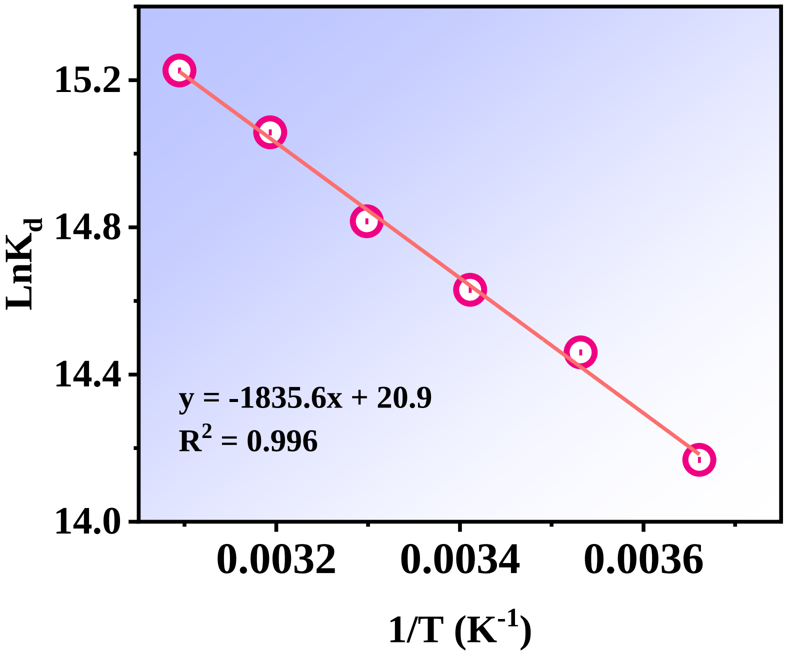

Fig. S18 The relationship between 1/T and LnK_d.

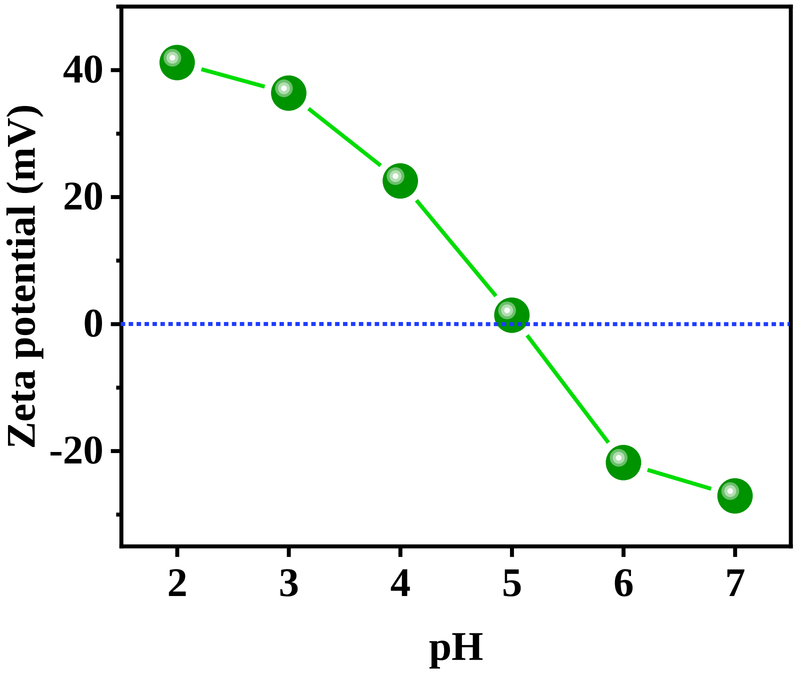

Fig. S19 Zeta potentials of CFeS aerogel at the pH value ranging from 2 to 7.

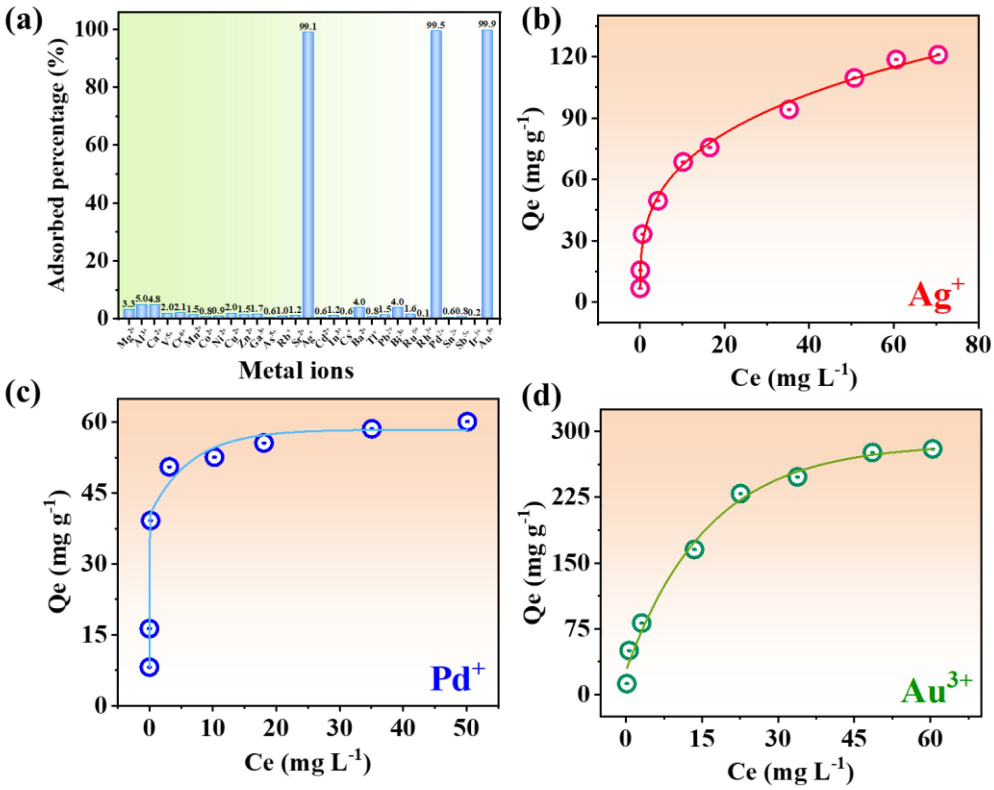

Fig. S20 Adsorption performance of CFeS aerogels after 90 days of storage in ambient air. (a) Adsorption selectivity of CFeS aerogels among 29 metal ions (100 ppb per ion). (b) Adsorption isotherm of Ag(I). (c) Adsorption isotherm of Pd(II). (d) Adsorption isotherm of Au(III).

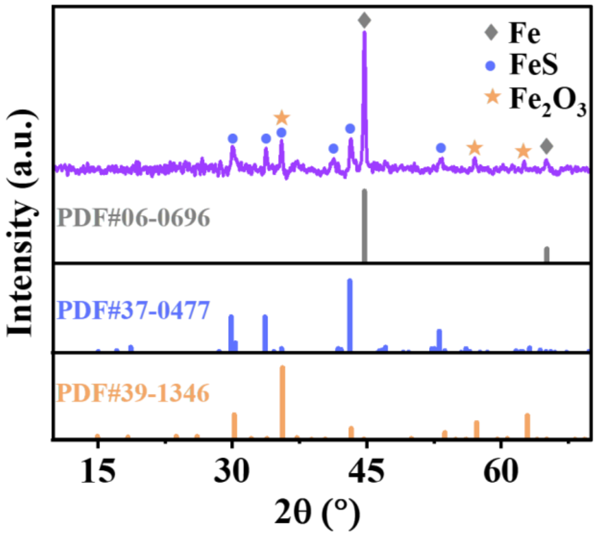

Fig. S21 XRD patterns of CFeS samples stored in air for 90 days.

Table S4 Cost analysis of laboratory-prepared CFeS aerogel.

Project		Unit price	Quantity/ Batch Cost	Cost estimation
Raw Material	I-carrageenan	\$30/kg	40 g	\$1.2
	$FeCl_3$	\$10/kg	40 g	\$0.4
	ethanol	\$2/L	2 L	\$4
	Ar gas	\$1/L	3 L	\$3
	H_2O	\$1/t	5 L	\$0.005
Equipment	stirring system	\$10000/set	\$10/use	\$10
	freeze dryer	\$30000/unit	\$30/use	\$30
	tube furnace	\$50000/unit	\$50/use	\$50
Energy	stir	\$0.1/kWh	0.75 h × 0.5 kW	\$0.0375
	freeze drying	\$0.1/kWh	36 h × 5 kW	\$18
	heat treatment	\$0.1/kWh	8.67 h × 10 kW	\$8.67
Labor		\$7/h	8h	\$56
total		-	-	\$18.11/g

Note: The laboratory-scale preparation produces 10 g of CFeS aerogel per batch.

Table S5 Production cost analysis of CFeS aerogel.

**Cost Category**	**Lab-Scale Cost**	**Pilot production cost**	**Description**
Raw materials	\$0.86/g	\$300/kg	Containing I-carrageenan, $FeCl_3$, ethanol, Ar gas, etc., the price of large-scale production can be further reduced by 50%
Equipment	\$9/g	\$300/kg	Scaled production improves equipment utilization and reduces the depreciation share
Energy	\$2.67/g	\$500/kg	After large-scale production, the average energy consumption is reduced
Labor	\$5.6/g	\$200/kg	Automated production reduces labor costs
Total	\$18.11/g	\$1.3/g	Lab-scale production costs are dominated by equipment depreciation and low yield rates, while scaled production costs will decrease significantly.

Table S6 Types and concentrations of metal ions in electronic wastewater, gold mine leachate, and electroplating wastewater.

**Wastewater type**	**e-Waste solution**		**CPU leachate**		**Simulated gold-ore leach solution**		**Electroplating wastewater**	
	Metal	concentration	Metal	concentration	Metal	concentration	Metal	concentration
**Composition**	Cu	351.9 ppm	Cu	242 ppm	Cu	21.2 ppm	Ag	200 ppm
	Ni	71.8 ppm	Ca	9.6 ppm	Fe	19.3 ppm	Cu	150.2 ppm
	Pt	3.1 ppm	Al	8.5 ppm	Au	1.7 ppm	Ni	75.3 ppm
	Au	1.9 ppm	Au	2.65 ppm	Ag	1.4 ppm	Zn	15.1 ppm
	Zn	1.7 ppm	Ba	620 ppb	Ni	0.8 ppm	Co	23.1 ppm
			Zn	200 ppb				
			Mg	170 ppb				
			Sn	106 ppb				
			Sr	70 ppb				
			Fe	68 ppb				
			Ni	54 ppb				
			Cr	13 ppb				
			Mn	13ppb				
			As	8 pbb				
			Pb	1 ppb				
**Reference**	[8]		[9]		[10]		[11]	

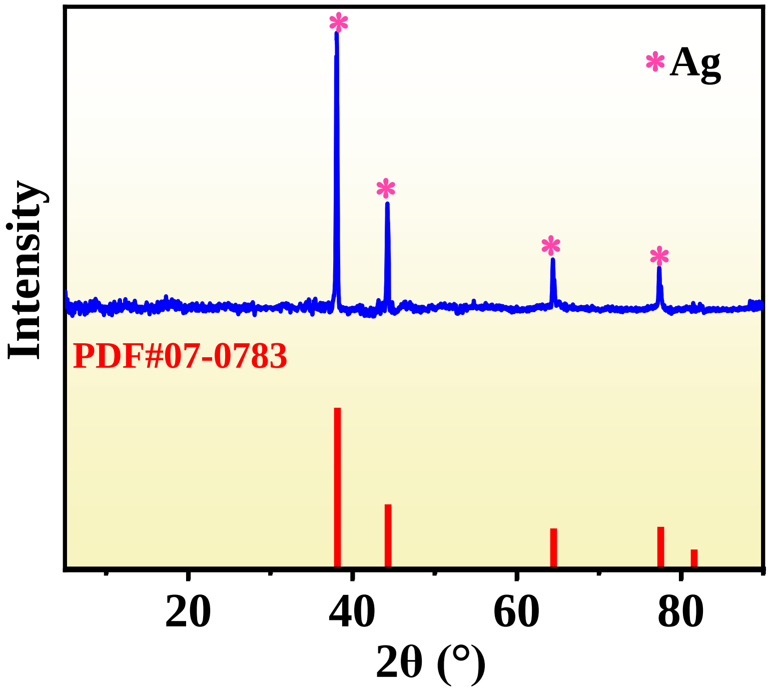

Fig. S22 XRD pattern of Ag recycled from wastewater.

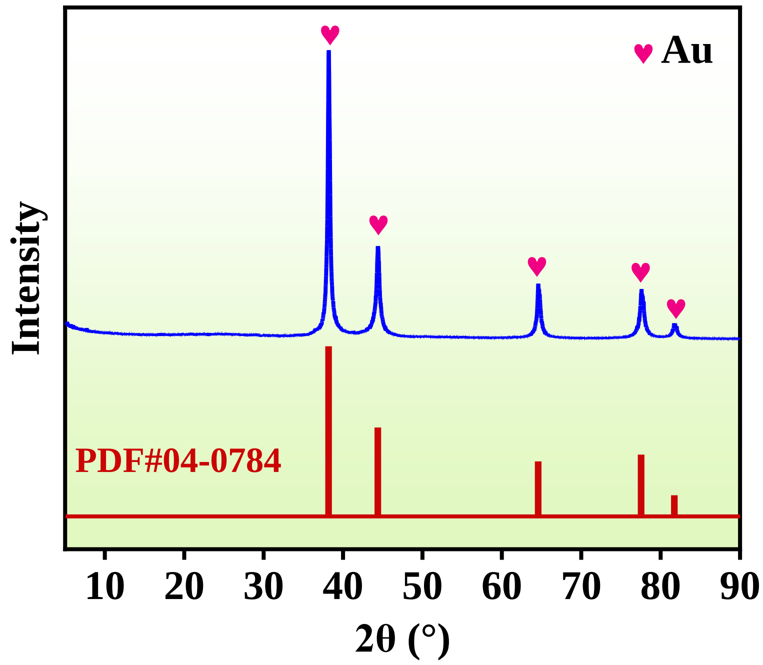

Fig. S23 XRD pattern of Au recycled from wastewater.

References

[1] Y.Y. Huang, Y.Y. Wu, W. Ding, Q. Sun, C. Hu, B.Z. Liu, H.X. Liu, H.L. Zheng, *J. Cleaner Prod*, **2022**, *339*, 130777.

[2] F. Wu, H. Li, Y. Pan, Y.H. Sun, J.M. Pan, *J. Hazard. Mater*. **2023**, *441*, 129917.

[3] Y.Q. Geng, J.Q. Li, W.J. Lu, N. Wang, Z.Y. Xiang, Y.Z. Yang, *Chem. Eng. J.* **2020**, *381*, 122627.

[4] M.H. Zhao, Y.F. Zhang, R.J. Yang, C. Wang, C. Xiong, H. Li, R.S. Zhu, S.X. Wang, Z.Y. Zeng, *Small Struct*. **2023**, *4*, 2300039.

[5] T. Xu, R.J. Qu, Y. Zhang, C.M. Sun, Y. Wang, X.Y. Kong, X. Geng, C.N. Ji, *Chem. Eng. J.* **2021**, *410*, 128225.

[6] C.Q. Mu, L. Zhang, X.M. Zhang, L.L. Zhong, Y. Li, *J. Hazard. Mater.* **2020**, *381*, 120943.

[7] H.E. Rizk, N.E. El-Hefny, *J. Alloys Compd.* **2020**, *812*, 152041.

[8]X.H. Li, Y.L. Wang, J. Wen, L.L. Zheng, C. Qian, Z.H. Cheng, H.Y. Zuo, M.Q. Yu, J.Y. Yuan, R. Li, W.Y. Zhang, Y.Z. Liao, Nat. Commun, 2023, 14, 263.

[9] F. Li, J.Y. Zhu, P.Z. Sun, M.R. Zhang, Z.Q. Li, D.X. Xu, X.Y. Gong, X.L. Zou, A.K. Geim, Y. Su, H.M. Cheng, Nat. Commun, 2022, 13, 4472

[10] S.R. Cotty, A. Faniyan, J. Elbert, X. Su, Nature Chemical Engineering, 2024, 1, 281–292.

[11] P.H. Shao, Z.W. Chang, M. Li, X. Lu, W.L. Jiang, K. Zhang, X.B. Luo, L.M. Yang, Nat. Commun, 2023, 14, 1365.
